# Trophic-level dependent concentrations of forever chemicals in insects of pristine areas are linked to morphological changes

**DOI:** 10.1038/s42003-026-10586-1

**Published:** 2026-07-09

**Authors:** Veronika Rosa Hierlmeier-Hackl, Patrick Krapf, Sabrina Gurten, Carolin Margit Strutzmann, Valentin Titus Schlenz, Timotheus Kopf, Wolfgang Moche, Korbinian Peter Freier, Florian Michael Steiner, Birgit Christiane Schlick-Steiner

**Affiliations:** 1 Department of Ecology, University of Innsbruck, Innsbruck, Austria; 2 Bavarian Environment Agency, Augsburg, Germany; 3 Organismal & Evolutionary Biology Research Programme, University of Helsinki, Helsinki, Finland; 4 Department of Evolutionary and Population Biology, Institute for Biodiversity and Ecosystem Dynamics, University of Amsterdam, Amsterdam, The Netherlands; 5 Environment Agency Austria, Vienna, Austria

**Keywords:** Entomology, Ecology, Chemical biology

## Abstract

Persistent, bioaccumulative and toxic chemicals (PBTs) are notoriously harmful, but their impact on insects in remote areas remains insufficiently explored. We investigate effects of PBTs on insects at remote high elevations in the European Alps, using herbivorous bumblebees, omnivorous ants, and necrophagous beetles – species of three trophic levels. We detect 30 PBTs in the insects’ bodies, including mercury, brominated flame retardants, polychlorinated biphenyls, and per- and polyfluoroalkyl substances. The concentrations increase from herbivores to omnivores to necrophages. For mercury, the concentrations are even 120-fold higher in carrion beetles than in bumblebees. Individual PBTs correlate positively with fluctuating asymmetry values (FA) in all species, representing morphological change and suggesting reduced fitness. Inbreeding values, as alternative cause of FA, are low and unlinked to FA. Nevertheless, correlations become non-significant for certain combinations of species and substances when including measurement uncertainty using Monte Carlo simulations. The impact of specific PBTs on FA must therefore be considered carefully, and measurement precision is still a limiting factor for field studies. Our data highlight the exposition and accumulation of PBTs along trophic levels in insects of pristine areas and evidence detrimental effects. We conclude that PBTs are likely a driving factor of today’s insect decline.

**Figure:**
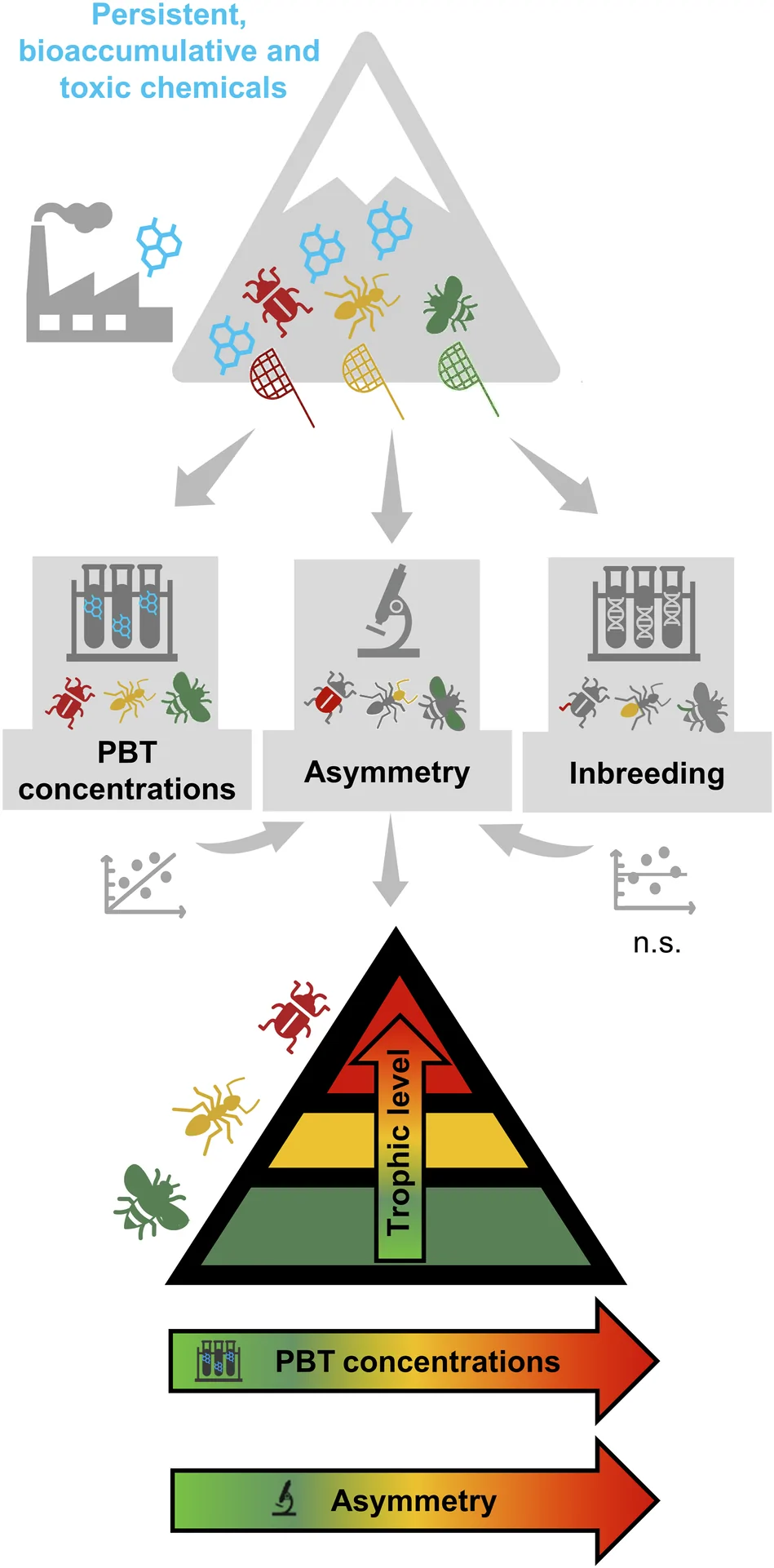

## Abstract (web summary)

Persistent, bioaccumulative and toxic chemicals (PBTs) accumulate along trophic levels of insects from remote areas of the Alps. Some PBT concentration levels correlate with fluctuating asymmetry as proxy for insect fitness.

## Introduction

Insects are major players in the biosphere. Besides the cohesion of whole ecosystem communities, insects ensure degradation of organic matter, biocontrol of pests, and pollination. As a key component of any food web, they bridge aquatic and terrestrial food webs^1^, as well as primary producers and consumers across trophic levels^2^. However, long-term data confirm global downward trends in their abundance, biomass, and species numbers^3–5^. This decline may have cascading effects for entire ecosystems^1^.

Habitat loss through urbanisation and intensification of agriculture is considered the main driver of the worldwide insect decline^6^. Climate change and biological factors such as invasive species and pathogens as well as environmental pollution through fertilisers and synthetic pesticides contribute also to decreasing insect populations^7^. However, the decline of insects is also documented in remote, semi-natural, and protected areas^3–5^. Hence, it is likely that further factors are decisive as well, such as lacking of habitat connection, leading to a reduced genetic exchange with other populations^8^. Additionally, atmospherically introduced substances, such as nitrogen compounds or long-range transported pollutants, must be considered. Nitrogen compounds lead to a fertiliser effect that modifies plant community compositions. This leads to changes in the food supply particulary for specialised insect species^9^. In the context of long-range transported pollutants, so far little attention has been paid to persistent, bioaccumulative, and toxic chemicals (PBTs), released anthropogenically into the environment from agricultural and industrial sources. Due to their high persistence as well as semi-volatile properties, PBTs are distributed via long-range atmospheric transport globally^10^. Through successive condensation and volatilisation processes, they accumulate in cold environments, such as in arctic and mountainous areas^11^. Historically, many aquatic and terrestrial environments were PBT-sinks. Rising global temperatures lead to greater volatilisation rates, and PBT-sinks turn into secondary sources from which PBTs are now reentering the global food webs^10,12,13^. PBTs include organic substances, containing various substance groups. Specifically, they comprise brominated flame retardants (BFRs)^14^ which are used in a broad range of products; organochlorine pesticides (OCPs), such as DDT, used for pest control^10^; polychlorinated biphenyls (PCBs), used as electrical insulating fluids^15^; and per- and polyfluoroalkyl substances (PFASs), used among other to coat textiles or packaging^16^. Since 2004, the production and use of 35 organic PBTs have been restricted through an international agreement, the Stockholm Convention^17^. Besides organic compounds, PBTs also include some heavy metals which behave similar to organic pollutants due to their persistence, bioaccumulation, and toxicity^18^. In this context, mercury is of special interest, because it is semi-volatile like organic PBTs, and released in huge quantities through goldmining and fossil fuel combustion^19^.

The ecotoxicological effects of PBTs on organisms are manifold. Carcinogenesis, endocrine disruption, damage of the immune system, and reduced reproductivity have been documented in various biota^20^. Specifically, for invertebrates, increased mortality, altered behaviour, and changes in sex ratio and developmental time have been detected^21^. Morphological changes, such as fluctuating asymmetry (FA; i.e., small differences between the left and right sides due to imprecisions in developmental processes^22^), have also been reported as a consequence of environmental stress through PBTs^23–25^. FA is therefore considered as effective to detect environmental stress in natural environments^24,25^.

Due to their lipophilicity, PBTs are stored in the fatty tissue of organisms^10^. They accumulate if the uptake rate through consumption is higher than the reduction through excretion, metabolism, and growth dilution^26^. The bioaccumulation in individuals leads to a subsequent biomagnification across food webs, and even low environmental concentrations of PBTs can have devastating effects on higher trophic levels as the concentrations increase with trophic position^27–29^. The bioaccumulation of PBTs is well documented for aquatic food-webs, including plankton-feeding species, several consumer ranks in fish, piscivorous bird species, seals, cetaceans, and semiaquatic predators such as polar bears. Here, the influence of various factors on the trophic transfer, the bioaccumulation patterns, the distribution along food chains, as well as the impact on marine predators as endconsumers are well studied^27,28,30,31^. Risk analyses for PBTs in aquatic organisms have been conducted for years, facilitating long-term studies and risk assessment^32^. In contrast, limited data exist on bioaccumulation within terrestrial food webs, particulary regarding terrestrial insects of different trophic levels as integral components of the food web^33^. Here, the number of studies on the trophodynamics such as the transfer of PBTs across trophic levels, the biomagnification and degradation is low^34^. Previous research focused on laboratory studies, and insects at natural or near-natural conditions are particularly understudied^21^. This is mainly due to the problem that PBTs occur in trace-levels in natural habitats, which renders quantification analytically demanding. Thus, the hitherto low number of field studies often focussed on insects from heavily contaminated sites where high body concentrations are expected^35–37^, whereas remote areas with lower and unknown pollution are underrepresented^21^. Generally, sample masses of individual insects are too little to be processed with state-of -the-art analyses to quantify concentrations of PBTs. Furthermore, standard methods for collecting insect masses like Malaise traps are not suitable for chemical trace-analysis because of cross-contamination. Hence, field-work to gather sufficient, species-specific insect biomass while respecting the high-quality demands of chemical trace-analytics is challenging.

In our study, we address this research gap on the accumulation of PBTs in insects as a potential cause of insect decline in remote natural habitats. We hypothesise that PBTs (1) abound in insects from remote natural habitats, (2) bioaccumulate with increasing trophic position, and (3) affect the insects´ morphology. To address (1) and (2), we assessed the PBT concentrations of terrestrial insects of three trophic levels (herbivorous bumblebees, omnivorous ants, and necrophagous carrion beetles) at two remote study areas in the Austrian Alps (Hoher Sonnblick) and German Alps (Zugspitze). Concentrations of PBTs in the bodies were measured in pooled samples by a chemical trace-analysis and related to the diet, i.e., trophic levels of the insects and isotopic characteristics. To address (3), we measured FA of the insect bodies (heads or wings). Inbreeding can also cause FA^24,25^, and we thus additionally assessed the insect populations genetically. By correlating concentration levels of PBTs and FA, we investigated whether higher concentrations of PBTs in the bodies resulted in higher asymmetry. Since PBTs encompass very many chemicals, we focused on substances regularly detected in the ambient-air and deposition of alpine environments^38^, especially BFRs, mercury, PCBs, OCPs, and PFASs.

This study aims to contribute to a better understanding of the rapid global insect decline, even in remote areas. We found that PBTs accumulate along trophic levels of insects from remote areas of the European Alps and that some PBT concentration levels correlate with FA as proxy for insect fitness. We therefore conclude that PBTs are further stressors contributing to the decline of insect populations.

## Results

We collected 331 bumblebees (herbivores), 440 ants (omnivores), and 239 carrion beetles (necrophages) for the chemical, morphological and genetic analyses. Of these, 209 were *Bombus pratorum* and 122 were *Bombus wurflenii*, 300 *Formica aquilonia* and 140 *Formica exsecta* and 239 *Nicrophorus vespilloides*. Data from additional 7279 ants (estimated) and 51 *Nicrophorus* spp. were included in the chemical analysis (see Methods). The insects were prepared for chemical, morphological and genetic analyses following Hierlmeier et al.^25^

### Analysis of PBTs in insects

Generally, our methodology proved suitable for quantifying PBTs in pooled insect samples, while at the same time determining the exact species, assessing genetic characteristics, and FA. Addressing hypothesis (1) “PBTs are abound in insects from remote natural habitats”, we demonstrated all investigated insects as exposed to PBTs. Over all organisms, we found 30 of 79 substances in concentrations between 0.0039 and 97 µg/kg wet weight.

**Fig. 1:**
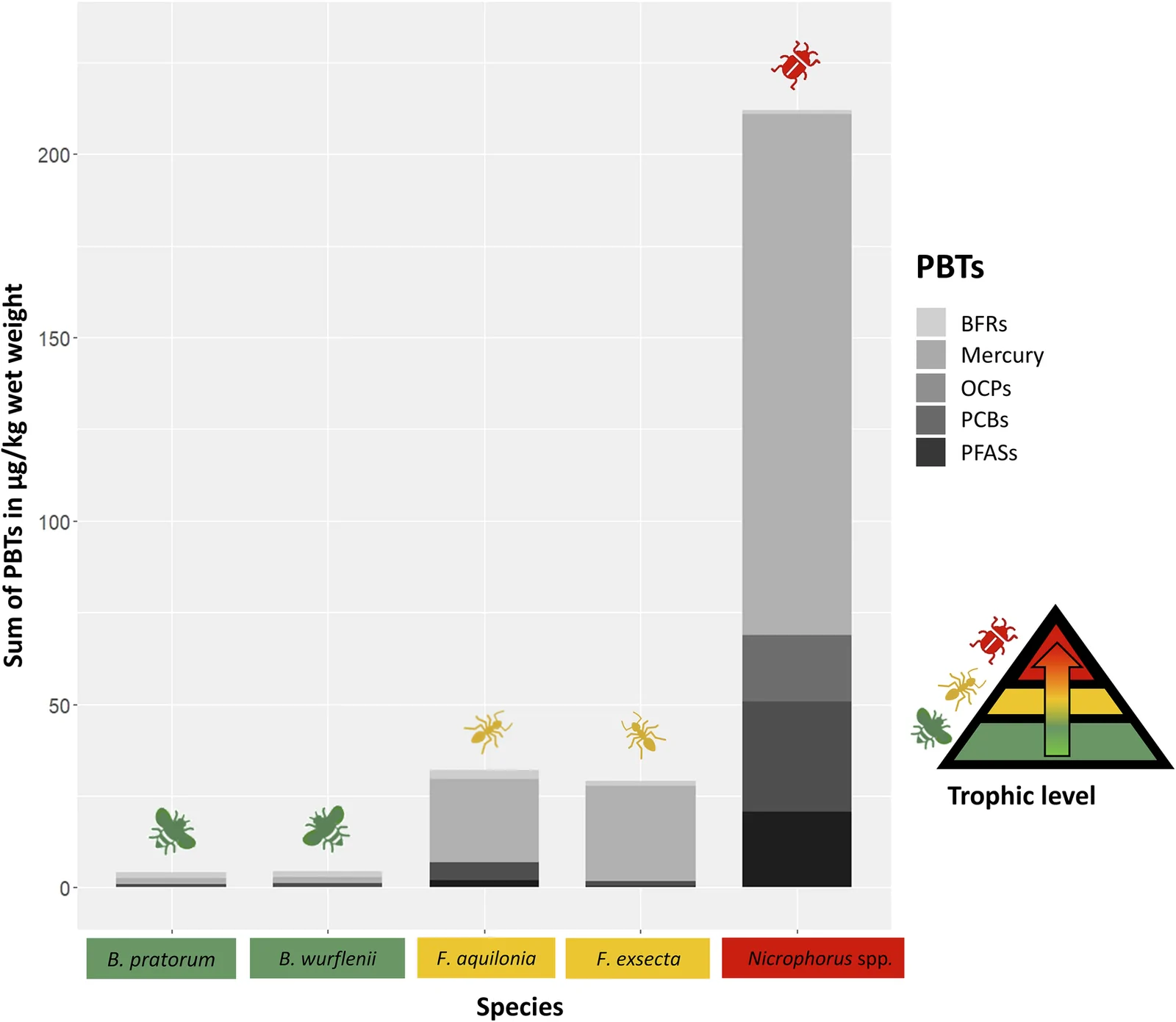
Concentrations of persistent, bioaccumulative, and toxic chemicals (PBTs) in insects of different trophic levels. PBTs in insect species (bumblebees, ants, carrion beetles) from three trophic levels at Hoher Sonnblick (Austrian Alps) and Zugspitze (German Alps). The concentrations consist of the added values of PBTs of a substance class, including both study sites (Supplementary Data 1). For the ants, a mean mercury value per study site was calculated with the values of the individual nests. For *Nicrophorus* spp., the values of mercury and PCBs consist of the mean values of 2019 and 2020 for Hoher Sonnblick. The classes of PBTs were brominated flame retardants (BFRs), mercury, organochlorine pesticides (OCPs), polychlorinated biphenyls (PCBs) and per- and polyfluoroalkyl substances (PFASs). The concentrations are depicted in µg/kg wet weight. llustrations were created in PowerPoint (Microsoft 365) with icons.

In the bumblebees, 25 of 46 investigated substances were detected (Supplementary Data 1A). BFRs were detected in concentrations between 0.0049 and 0.86 µg/kg wet weight (Fig. 1). Mercury concentrations ranged between 0.65 and 0.81 µg/kg wet weight. PCBs were detected in concentrations between 0.067 and 0.22 µg/kg wet weight. PFASs were not detectable in the bumblebee samples.

In the ants, 20 of 46 PBTs analysed were detected (Supplementary Data 1B). BFRs were detected in concentrations between 0.0067 and 1.2 µg/kg wet weight (Fig. 1). The concentrations of mercury ranged between 5.3 and 27 µg/kg wet weight. PCBs were detected in concentrations between 0.10 and 0.47 µg/kg wet weight. PFASs (PFHxA, PFNA, PFOS) were detected at the detection limit of 0.50 µg/kg wet weight.

For *Nicrophorus* spp., 79 PBTs were analysed, as sufficient biomass was available, and 31 were detected (Supplementary Data 1C). The concentration of BFRs ranged between 0.0039 and 0.56 µg/kg wet weight (Fig. 1). The concentrations of mercury were between 45 and 97 µg/kg wet weight. OCPs with concentrations between 1.10 and 7.60 µg/kg wet weight were detected and PCBs with concentrations between 0.14 and 12 µg/kg wet weight. The concentrations of PFASs (PFDA, PFDoDA, PFNA, PFHxA, PFHxS, PFOA, PFOS, PFUnDA) ranged from below the limit of quantification of 0.50 to 12 µg/kg wet weight.

### PBT concentrations and trophic levels

Addressing Hypothesis (2) “PBTs bioaccumulate with increasing trophic position”, we found that the total sum of PBTs increased from bumblebees to ants to carrion beetles (Fig. 1). Considering the concentrations of BFRs, mercury, PCBs and PFASs we found that certain PBTs accumulate with increasing trophic level.

**Fig. 2:**
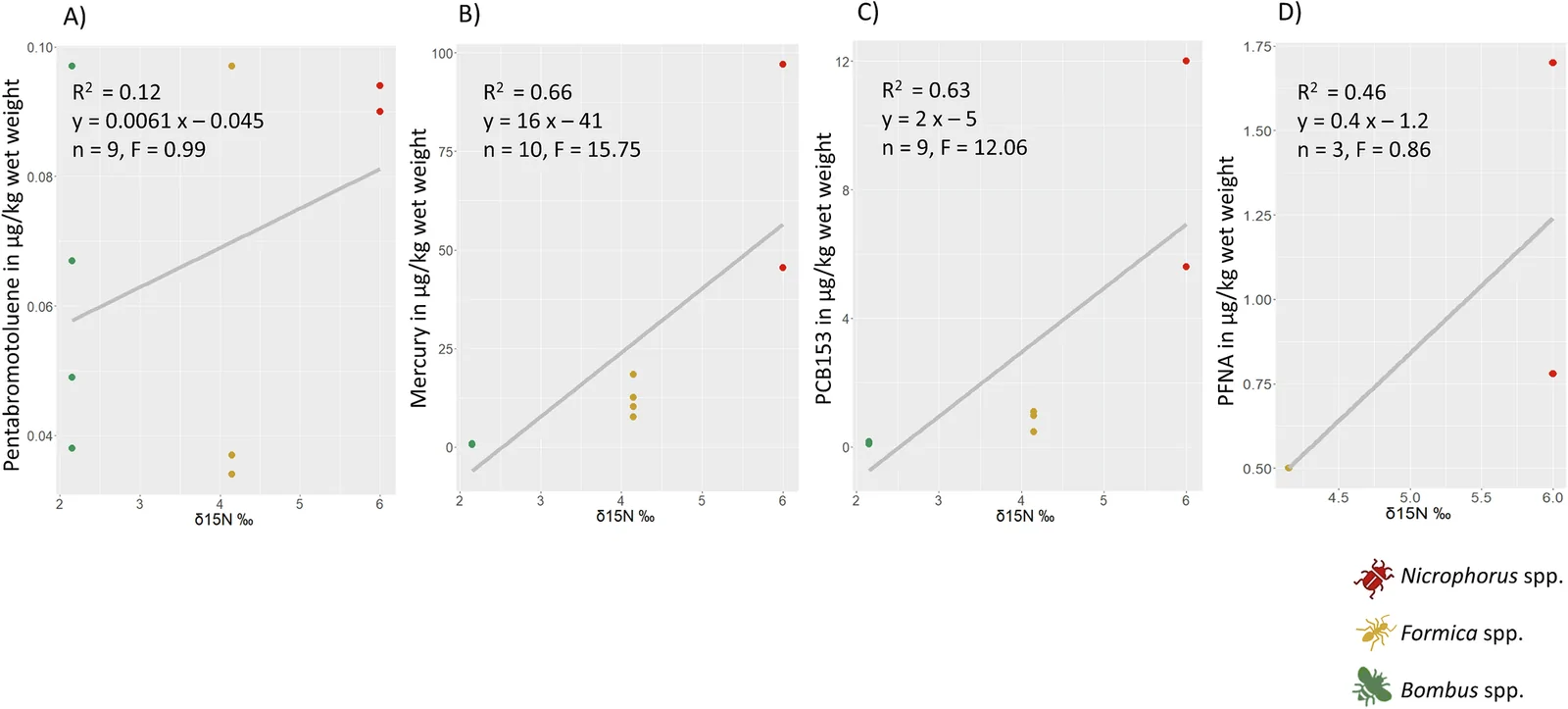
Correlation of concentrations of pentabromotoluene, mercury, polychlorinated biphenyl 153 (PCB153), and perfluorononanoic acid (PFNA) with isotopic data as a proxy of trophic position. Correlation of the flame retardant pentabromotoluene (**A**), mercury (**B**), the polychlorinated biphenyl PCB153 (**C**), the per- and polyfluoroalkyl substance PFNA (**D**), and specific isotope values of δ15N from the literature found in insect species from three trophic levels at Hoher Sonnblick (Austria) and Zugspitze Germany) in µg/kg wet weight. *Bombus* spp. (*B. pratorum*, *Bombus wurflenii*) represent herbivores, *F. aquilonia* and *Formica exsecta* represent the intermediate trophic level as omnivores and *Nicrophoru*s spp. as necrophages represent the high-end consumer level. Each datapoint represents the regional concentrations of the substance in the samples from Hoher Sonnblick and Zugspitze. Please note that both axes differ across PBTs. llustrations were created in PowerPoint (Microsoft 365) with icons.

In the case of BFRs, represented by pentabromotoluene, *F. exsecta* and *B. wurflenii* had the highest values. The concentrations in *Nicrophorus spp*. were similarly high whereas *F. aquilonia* had the lowest values (Supplementary Data 1). A pattern of enrichment, using nitrogen stable-isotopes values as proxy for trophic levels, was not found (from the literature, *n* = 9; *p* = 0.3529, Fig. 2A).

Compared with the concentration of mercury in the bumblebees (herbivores), the maximum concentration in ants (omnivores) was 33-fold higher. *Nicrophorus* spp. (necrophages) had a more than 5-fold higher body burden of mercury than the ants and a more than 120-fold higher body burden than the bumblebees (Supplementary Data 1). The concentrations of mercury increased with increasing nitrogen stable-isotopes values as proxy for trophic levels (from the literature; *n* = 10; *p* = 0.004, Fig. 2B).

The enrichment across trophic levels was also detected for the PCB153. Compared with the concentration of PCB153 in the bumblebees (herbivores), the concentration in ants (omnivores) was higher by a factor of 6.5. *Nicrophorus* spp. had a 12- fold higher body burden than the ants and a more than 70-fold higher body burden than the bumblebees (Supplementary Data 1). The concentrations of PCB153 increased with increasing nitrogen stable isotopes values (from the literature; *n* = 9; *p* = 0.010, Fig. 2C).

Considering PFASs, we focused on PFNA due to its high environmental risk, regarding the high toxicity potential^39^. PFASs in general, where not detected in the bumblebee at all. For ants, the concentrations of PFNA were at the quantification limit, while the maximum of PFNA for *Nicrophorus* spp. was more than 3-fold higher (Supplementary Data 1). At *p* = 0.5235, an interpretation of the model was not possible (*n* = 3; Fig. 2D).

A Spearman’s Rank correlation, where trophic levels were represented by ranks, confirmed the pattern of enrichment with trophic levels solely for PCB153 (ρ = 0.94; *p* = 0.00017), whereas a significant positive correlation was not found for pentabromotoluene (ρ = 0.10; *p* = 0.79), Hg (ρ = 0.94; *p* = 0.06) nor PFNA (ρ = 0.87; *p* = 0.33).

### Asymmetry in insect bodies

For both bumblebee species, left and right forewing shapes differed significantly (Procrustes ANOVA; *p* < 0.0001, Supplementary Table 1). The FA-scores in the bumblebees ranged between 0.009 and 0.029 (Supplementary Table 1, Supplementary Data 2A).

The Procrustes ANOVA for ants differed significantly for the left and right side of the heads of *F. aquilonia* and *F. exsecta* (Procrustes ANOVA; *p* < 0.0001, Supplementary Table 1). The FA-scores in the ants ranged between 0.004 and 0.032 (Supplementary Table 1, Supplementary Data 2B).

For carrion beetles, solely individuals of *N. vespilloides* were analysed morphologically, as additional individuals of *N. vespillo* were used to increase the biomass for chemical analysis. In the Procrustes ANOVA for carrion beetles, the asymmetry of the shape of forewings differed significantly for *N. vespilloides* (Procrustes ANOVA; *p* < 0.0001, Supplementary Table 1). FA values were between 0.025 and 0.077 and thus the highest values of all species (Supplementary Table 1, Supplementary Data 2C).

### Genotyping and inbreeding analysis

DNA was extracted and genotyped using six and eight microsatellite loci for *B. pratorum* and *B. wurflenii*, respectively; eleven and eight for *F. aquilonia* and *F. exsecta*, respectively; and twelve for *N. vespilloides*.

In *B. pratorum*, after correction for multiple comparison, four loci deviated from the Hardy-Weinberg-Equilibrium and one locus combination was significantly linked (polymorphism and heterozygosity in Supplementary Table 2). In *B. wurflenii*, three loci deviated from the Hardy-Weinberg-Equilibrium and no locus combination was significantly linked (polymorphism and heterozygosity in Supplementary Table 2). At a mean of 0.80 for *B. pratorum* and 0.82 for *B. wurflenii*, multilocus heterozygosity (MLH) values, used as an inbreeding measure, were consistently high in both species. The inbreeding coefficients (F_IS_) over all loci were close to zero (Supplementary Table 2).

In *F. aquilonia* and *F. exsecta*, no deviation from the Hardy-Weinberg-Equilibrium or alleles in linkage disequilibrium were found (polymorphism and heterozygosity in Supplementary Table 2). The mean MLH was 0.51 for *F. aquilonia* and 0.60 for *F. exsecta*. The inbreeding coefficients (F_IS_) over all loci were close to zero (Supplementary Table 2).

Due to deviation from the Hardy-Weinberg-Equilibrium for both sampling regions, five of 12 loci were excluded from the analysis for *N. vespilloides*. Two loci deviated significantly from the Hardy-Weinberg-Equilibrium after Bonferroni-Holm correction for the population of Zugspitze. Four loci were significantly linked (polymorphism and heterozygosity in Supplementary Table 2). The mean values of MLH were 0.73. The inbreeding coefficients (F_IS_) over all loci were also close to zero (Supplementary Table 2).

No influence of inbreeding on the FA values was found in any organism.

### Correlation of individual PBTs with fluctuating asymmetry (FA) values

To investigate Hypothesis (3) “FA increases with PBT concentrations over all species” we used simple linear regressions. Additionally, to investigate whether FA values correlate with specific PBTs within species, we used multiple linear regressions. To consider chemical measurement inaccuracy, we conducted Monte Carlo Simulations for the PBTs and linear models of these simulations in a second step.

**Fig. 3:**
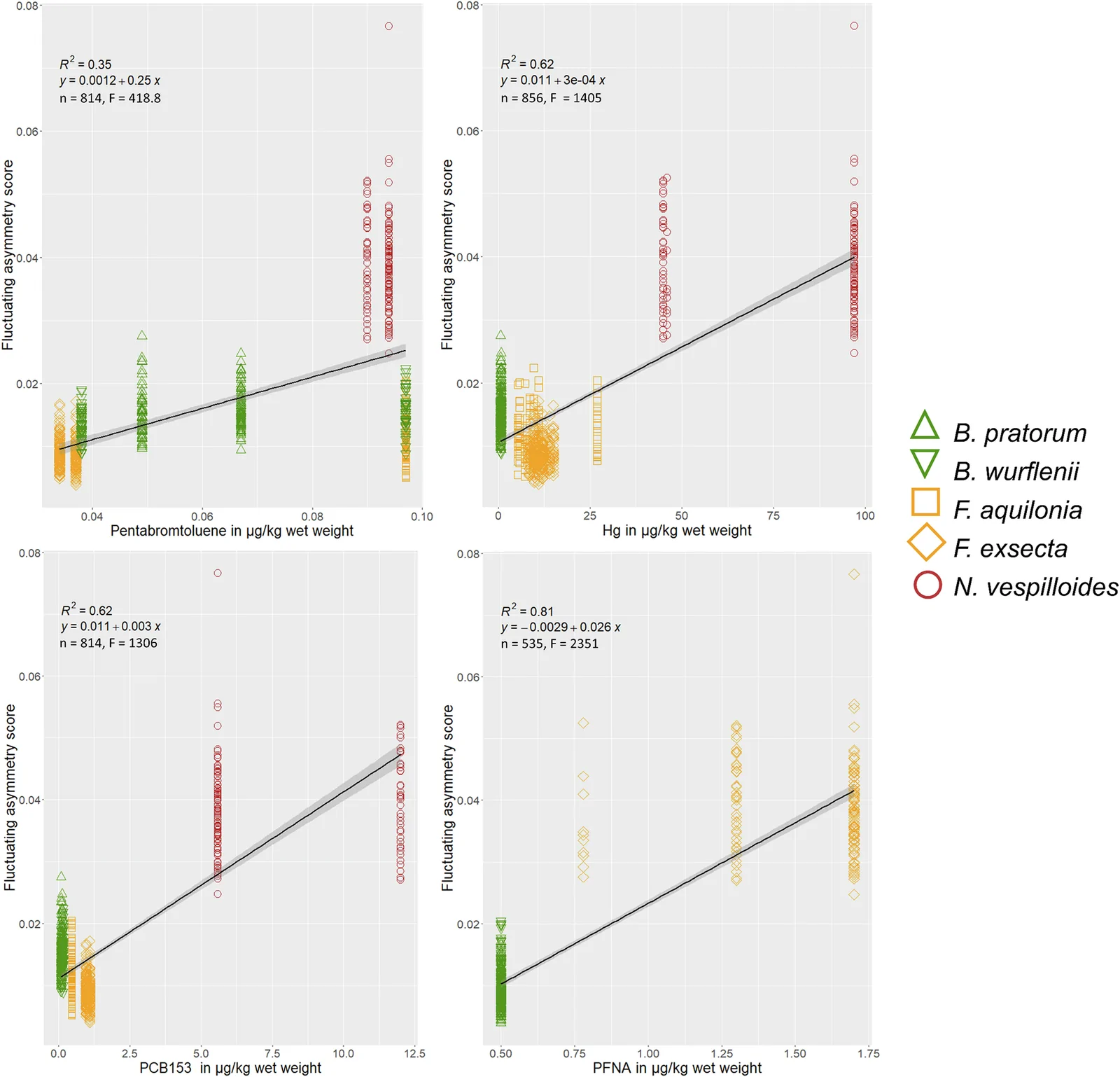
Correlations of fluctuating asymmetry (FA) scores with the concentrations of pentabromotoluene, mercury, polychlorinated biphenyl 153 (PCB153), and perfluorononanoic acid (PFNA) found in insect bodies. Correlations between the PBTs and fluctuating asymmetry scores of the individuals were found in *B. wurflenii, B. pratorum, F. aquilonia, F. exsecta* and *N. vespilloides* and fluctuating asymmetry scores of the individuals. The PBTs plotted were chosen as representatives of brominated flame retardants, heavy metal, polychlorinated biphenyls, and per- and polyfluoroalkyl substances. Please note that the x-axis differs across PBTs.

Over all species, the concentrations of pentabromotoluene, mercury, PCB153, and PFNA correlated positively with the FA scores (*p* < 0.001; Fig. 3). Pentabromotoluene explained 35% of the FA scores, mercury and PCB153 62%, and PFNA 81%. Of the remaining PBTs, eight BFRs, four PCBs, and one PFAS correlated positively with the FA scores, five BFRs and one PCB correlated negatively with the FA scores (*p* < 0.005, Supplementary Figs. 1–3). As OCPs were solely found in *N. vespilloides*, correlations across species were not examined.

Within species, the concentration levels of PBTs and asymmetry of the body-structures correlated positively for a relevant number of the investigated PBTs. However, most of these correlations vanished when assessing the results of our Monte Carlo Simulation, which we used to test the robustness of our results regarding the given measurement accuracy of the chemical trace-analysis (Table 1). Whereas all correlations between Anti-DP, DBDPE, BDE 207, BDE 209, PCB28, PCB 52, and PCB 101 with FA were no longer significant after the Monte Carlo Simulation, the correlations of BDE47, BDE85, BDE99, BDE100, BDE154, BDE196, and mercury remained significant. For pentabromotoluene, PCB138, PCB153, PCB180, the correlations with FA partly stayed significant and partly become non-significant, depending on the organism.

**Table 1:** Correlations between body concentrations of persistent, bioaccumulative, and toxic chemicals (PBTs) and fluctuating asymmetry (FA) scores

Organism	Linear model (z ~ ax + by + c)	a (slope MLH)	b (slope pollutant)	No. of models significant in testing of 1000 MC runs	significance (before MC)
*B. wurflenii*	forewing shape FA ~ MLH + pentabromotoluene	−1.48 × 10^−3^	46.55 × 10^−6^	674	*
	forewing shape FA ~ MLH + Anti-DP	−1.48 × 10^−3^	196.20 × 10^−6^	356	(*)
	forewing shape FA ~ MLH + DBDPE	−1.48 × 10^−3^	−91.54 × 10^−6^	230	(*)
	forewing shape FA ~ MLH + BDE47	−1.48 × 10^−3^	27.46 × 10^−6^	639	*
	forewing shape FA ~ MLH + BDE85	−1.48 × 10^−3^	1.62 × 10^−3^	675	*
	forewing shape FA ~ MLH + BDE99	−1.48 × 10^−3^	83.22 × 10^−6^	690	*
	forewing shape FA ~ MLH + BDE100	−1.48 × 10^−3^	376.20 × 10^−6^	797	*
	forewing shape FA ~ MLH + BDE154	−1.48 × 10^−3^	832.20 × 10^−6^	667	*
	forewing shape FA ~ MLH + BDE196	−1.48 × 10^−3^	832.2 × 10^−6^	659	*
	forewing shape FA ~ MLH + BDE207	−1.48 × 10^−3^	3.43 × 10^−3^	189	(*)
	forewing shape FA ~ MLH + BDE209	−1.48 × 10^−3^	5.28 × 10^−6^	676	*
	forewing shape FA ~ MLH + Mercury	−1.48 × 10^−3^	24.97 × 10^−6^	615	*
	forewing shape FA ~ MLH + PCB28	−1.48 × 10^−3^	137.32 × 10^−3^	66	(*)
	forewing shape FA ~ MLH + PCB52	−1.48 × 10^−3^	2.75	21	(*)
	forewing shape FA ~ MLH + PCB101	−1.48 × 10^−3^	−274.63 × 10^−3^	89	(*)
	forewing shape FA ~ MLH + PCB138	−1.48 × 10^−3^	59.70 × 10^−3^	736	*
	forewing shape FA ~ MLH + PCB153	−1.48 × 10^−3^	36.62 × 10^−3^	779	*
	forewing shape FA ~ MLH + PCB180	−1.48 × 10^−3^	72.27 × 10^−3^	800	*
*F. aquilonia*	head shape FA ~ MLH + pentabromotoluene	57.40 × 10^−3^	−22.76	19	(*)
	head shape FA ~ MLH + DBDPE	57.40 × 10^−3^	−359.29 × 10^−3^	66	(*)
	head shape FA ~ MLH + PCB28	57.40 × 10^−3^	−100.39 × 10^−3^	172	(*)
	head shape FA ~ MLH + PCB138	57.40 × 10^−3^	−620.59 × 10^−^^3^	110	(*)
	head shape FA ~ MLH + PCB153	57.40 × 10^−^^3^	−525.1 × 10^−3^	29	(*)
	head shape FA ~ MLH + PCB180	57.40 × 10^−^^3^	−1.37	39	(*)

Results of multiple linear models (z = ax + by + c) for *B. wurflenii* and *F. aquilonia* after taking into account detection errors of ±30% for organic PBTs and ±13% for mercury in chemical analysis via 1000 Monte Carlo Simulations (MC): Linear model with z = FA score of forewing shape or head shape, x = multilocus heterozygosity (MLH) value as genetic factor, y = concentration of PBT, c = intercept; * indicating *p* < 0.05.

For bumblebees, significant correlations were solely found in *B. wurflenii* between the measured concentrations of eleven BFRs, mercury, and the six PCBs and FA scores analysed (*p* < 0.05) before the Monte Carlo Simulation. After the Monte Carlo Simulation, the correlation between FA and seven BFRs, mercury, and three PCBs remained significant. Significant correlations occured in *F. aquilonia* for four PCBs and two BFRs before the Monte Carlo Simulation. After the Monte Carlo Simulation, the correlations expired. For *F. exsecta*, no correlation occured between FA and PBTs measurements. Although *N. vespilloides* had the highest concentrations of pollutants as well as the highest values of symmetries, no significant correlations occurred.

## Discussion

Insect decline, especially in remote areas, has not been linked to a single or few decisive factors. Instead, the underlying reasons likely are manifold. Here, we examined the prevalence, bioaccumulation, and impacts of atmospherically dispersed PBTs in insects of the European Alps. To our knowledge, our results indicate for the first time that even in pristine areas, insects are exposed to relevant levels of PBTs.

The PBTs, we have found in the insect samples, also occur in atmospheric deposition in our study areas^38^. Hence, our Hypothesis (1) is supported that PBTs are detectable in insects even though sources are spatially distant. It is striking that we still detected PBTs, such as Endrin and Heptachlor, even though their production, sale, and use have been banned by the Stockholm Convention since 2004^17^. At the same time, our findings strongly suggest that such conventions are effective, as many substances, such as Dieldrin and Mirex, which are now banned, were not detectable in any insect. However, we still detected recently restricted PBTs, from the class of PFASs such as PFHxS. Since the restriction of PFOS in 2006, PFHxS was produced in large amounts as a substitute for PFOS until 2017^40,41^. This indicates that insects accumulate PBTs that have been used for a long time but also react particularly quickly and sensitively to novel substances. The analysis of an even more complete array of PBTs in our study was impractical financially. To develop a more complete picture of exposure to environmental pollutants of wild alpine insects, additional studies will be needed that include further PBTs such as UV-328, and heavy metals, such as lead and cadmium.

Comparing our data with PBT concentrations in non-alpine bumblebees, ants, and carrion beetles is difficult because data for the latter are scarce. Solely, Helms and Tweedy^42^ reported higher concentrations in three species of invasive fire ants with concentrations of mercury between 33.6 and 63.6 µg/kg body mass. For two species in their study, the concentrations of 21.3 and 25.9 µg/kg were closer to our findings between 5.3 and 27 µg/kg wet weight.

BFRs, mercury, and PCBs occured in all organisms. PFASs were exclusively found in ants and carrion beetles, although they have been used over years in various consumer products and were found in several terrestrial and aquatic biota^16^. It is possible that PFASs were accumulated by the bumblebees as well, but the concentrations were below our detection limits. We assume the same effect for the class of OCPs, which where detectable only in carrion beetles. In addition, preliminary studies have shown that OCPs were solely detectable in carrion beetles^43^.

We assume that several factors, such as life history traits, affect the accumulation of PBTs in insects. Yung et al.^44^ demonstrated that the enrichment of PBTs, such as mercury, is at its highest for insect species with almost permanent contact with the soil. For example, mercury in the soil penetrates the cuticle at prolonged dermal contact^35^. Carrion beetles and ants spend more time in direct contact with soil than bumblebees, and we cannot exclude that this increases the concentrations of PBTs in their bodies. The concentrations of PBTs within the bumblebee species are also different, and the higher concentrations in *B. wurflenii* can possibly be attributed to their mostly underground nesting. In addition, it must be considered that species may have different metabolism and excretion rates^26^, leading to varying reduction rates in the body. Therefore, detectable concentrations in the bodies could vary in the case of similarly high exposure through dermal contact.

Aditionally, lifespan can affect concentrations. For birds, marine mammals and fish, studies demonstrated that concentrations of PBTs increase with the age of individuals^45^. Workers of bumblebees have a lifespan of a few weeks, so our data represent a snapshot, while the ants and burying beetles in our study live for around one year and longer^46,47^. It is also assumed that the increased lipid content in insects of higher trophic positions, such as predatory insects, increases enrichment because PBTs are highly lipophilic^48^. In addition, high bioaccumulation rates of PBTs can positively correlate with higher n-octanol water partition coefficients (log KOW) that lead to lipid solution^27^. If an organism’s fat tissue is rapidly depleted, such as through food scarcity, PBTs can further be concentrated to higher levels in the organism’s remanining biomass^49^. The reduction in insect mass during metamorphosis, combined with a lack of feeding, also causes a concentration effect and increases the burden on the organism^50^.

Nevertheless, we consider the accumulation via the trophic cascade as the main reason for the different concentration values in different insect species. Studies confirm the impact of diet on PBT accumulation in organisms^51,52^, corroborating our Hypothesis (2). Biomagnification^51^–increasing concentration with increasing trophic level – applies to whole food webs^27,29–31^. For example, Goerke et al. demonstrated a 30 to 160-fold biomagnification of PBTs in top predators such as elephant seals relative to krill^53^. In our study, we demonstrated an increase in the mean sum of concentrations of PBTs within terrestrial habitats for insects along trophic levels. This finding is consistent with, for example, that of Riva-Murray et al. who demonstrated three times higher levels of methylmercury in predatory aquatic insects than in primary consumers and significant correlations between the concentrations of methylmercury and their trophic positions, indicated by δ15N values^54^. In our study, literature data of stable-isotopes δ15N were used as proxies for trophic positions of the species investigated. Such data are scarce, unfortunately, in particular for the geographical regions and the altitudes investigated in our study. The results differed for one PBT when replacing the δ15N values with ranks in the Spearman’s rank correlation. This suggests that the literature data of stable-isotopes δ15N, used in our study, are imperfect as proxy for assessing that potential PBT accumulation with increasing trophic level. Therefore, we suggest that using literature data of stable-isotopes δ15N should be regarded as a starting point but that in future projects empirically measured δ15N of the respective insects should be included as a more robust proxy for trophic level.

The biomagnification in terrestrial food webs is less investigated, and biomagnification processes of aquatic systems cannot be completely transferred to terrestrial ecosytems^36^. Nevertheless, Liu et al.^36^ demonstrated the biomagnification of PCBs and halogenated flame retardants per trophic level within a terrestrial insect-dominated food web. As each trophic transfer holds the potential for biomagnification, higher concentrations of PBTs correlate with higher trophic positions in the food web^55^. Li et al. also demonstrated a more than 2-fold higher concentration of mercury in omnivorous bugs than in primary consumers, such as butterflies^56^. Liu et al. found higher levels of BFRs and PCBs in predators such as dragonflies than in primary consumers such as grasshopers and bugs^37^. We obtained similar results: The strong increase in mercury concentrations from bumblebees to ants to carrion beetles was striking. This can be explained by the different trophic levels of investigated species and their specific exposition to PBTs via food items. Bumblebees are exposed to mercury through pollen and nectar of plants^57^. Plants accumulate mercury from atmospheric deposition and the storage in the plant biomass increases with the concentrations of mercury in the soil^58^. As omnivores, ants also consume plant-based food. Aditionally, mercury accumulated by prey animals^35^, and mercury found in the honeydew of aphids^59^ leads to higher concentrations in the diet of ants. As necrophages, carrion beetles are on a higher trophic level than ants and bumblebees. Studies demonstrate the high levels of pollutants in carcasses of wild animals^60^, on which carrion beetles feed. Albinska et al.^60^ found concentrations of 20 µg/kg in the muscles and up to 80.2 to 128.5 µg/kg mercury in the kidneys of deers, roughly in the range we found in the carrion beetles (45 to 97 µg/kg wet weight). As carrion beetles subvert the animal carcasses and bury them in the ground for the brood, pollutants can also enter the body through contact with food. The high concentrations in their food may also explain why we found some PBTs solely in carrion beetles, such as DDE, a metabolite of the insecticide DDT. The harmful effects of DDT on burying beetles were already recorded in the early application phase^61^.

It can be assumed that several factors, such as life history traits and metabolism, affect the accumulation simultaneously. However, it is unclear how the individual factors interact and affect the accumulation. Studies, under controlled conditions, such as laboratory studies with known concentrations of PBTs in the food and soil, as well as known age of the insects, could provide further information (also see three paragraphs further down).

PBTs and FA were correlated for body structures of vertebrates^23^. For insects, morphological changes in body structures as a consequence of PBT bioaccumulation were also demonstrated^25^. FA is considered as a proxy for disturbances during development, triggered, for example, by genetic bottlenecks, overwintering stress, or harmful chemicals^62^. Therefore, FA has been used as fitness proxy as individuals with higher symmetry have advantages in, for example, mating^63^. In addition, more symmetric idividuals can have a longer life span because of lower susceptibility to biological factors, such as fungal infestation, whereas insects with asymmetrical wings are more frequently eaten by predators and are restricted in foraging, due to restricted flight performance^63^. Benítez et al.^64^ demonstrated that wing shape asymmetry is a simple, cost-effective, and reliable method for monitoring impacts of chemicals on organisms and morphological changes in body structures as a consequence of environmental chemicals.

We found an increase of FA with increasing concentrations of certain BFRs, mercury, PCBs, and PFASs over all investigated insects, supporting Hypothesis (3).

Nevertheless, a significant, positive correlation between the concentrations and the FA did not occur for all PBTs, and our results indicate that the impact of specific PBTs on FA must be considered in more detail, for example within individual insect species. We solely found correlations between the FA of *B. wurflenii* and *F. aquilonia* and certain PBTs. For the best of our knowledge, we here were the first to include measurement uncertainty in the analysis of FA, as a consequence of measurement uncertainties in trace analysis of PBT concentrations in biota. As the significant correlations for *F. aquilonia* disappeared after including the measurement uncertainty in the linear models with Monte Carlo Simulation, while correlations for *B*. *wurflenii* remained, the necessity of including precision of measurements as a limiting factor in the analysis is confirmed. Furthermore, correlations for *B. wurflenii* remained only for certain PBTs, which further reinforces the robustness of our results for these substances but not the others. This result indicates as well that even though we invested a lot of effort in gathering enough sample mass, we are still confronting an analytical limit concerning the accuracy of chemical trace-analysis.

Although concentrations of PBTs and FA values were generally higher in ants and carrion beetles, significant correlations were absent. A possible explanation might be that organisms react differently to PBTs^65^. It is possible that *B. wurflenii* is particularly susceptible to expressing FA at the detected concentrations of PBTs. Dobrin and Corkum found no correlation between FA and PCBs in mayflies and assumed that the stress from PBTs was not strong enough to trigger FA in these species^66^. Mielczarek et al. demonstrated FA in Hymenoptera triggered by heavy metals^67^. As we found no correlation for *B. pratorum*, we assume that this species is more resistant to PBTs. Variations in species’ susceptibility to toxicants, even at the intraspecific level, are known to occur, due to, for example, genetic factors, age, circadian rhythm, and biotic interactions^68^. In future research, field studies should be supplemented with laboratory studies, administering a known dose of PBTs, to investigate dose- and substance-dependent effects in different insect species and to exclude biotic factors. Alternatively, it is conceivable that the body structures used for quantification of FA in ants and carrion beetles react less sensitively to accumulated PBTs. This may be attributed to the more resilient physiology of these organisms in response to toxins. Carrion beetles, for example, have developed strategies to neutralise toxins in their food source. They inoculate carcasses with beneficial microbes from their own intestines. This causes a biochemical change that inhibits the growth of toxic polyamines and promotes the development of burying beetle larvae^69^. Burying beetles accumulate PBTs but other factors may, in fact cause high asymmetry. In our study, we excluded inbreeding, but other studies suggest that hormones and parasites can also lead to FA^70,71^. This confirms the necessity of investigating other triggers of FA in future studies. Additionally, other body structures should be investigated. Chang et al. demonstrated that toxicants trigger FA in one specific body structure, while others were not affected^72^. In future studies, hindwings and parts of the legs, for example, should be considered, too. Although FA is considered as a reliable method for monitoring developmental disturbances in insects, in some studies no correlation between the fitness of insects and FA was found^73,74^. Therefore, in future studies, other fitness proxies should be investigated additionally, such as individual biomass. Studies demonstrate decreased biomass in individuals exposed to PBTs. Another possible explanation for missing correlations might be a higher mortality at early development stages of the individuals with high FA values^75^. For our study, this would mean that insects with high FA values, triggered by PBTs, could not be collected due to their early death. Finally, although this study covered various PBTs, future studies should include additional substances, such as cadmium.

For many potential drivers of insect decline, data are limited or the setting in field studies is too complex. PBTs can lead to altered reproduction including a lower number of eggs, hatch rates as well as decreased development and survival rates of the offspring. Therefore, PBTs can severely affect natural insect populations^76,77^. With our study, we provided reliable data on one driver. Although other drivers still need to be examined more closely, we contributed to the understanding of the global decline. We identified PBTs as a potential risk for insects in remote areas. Although additional studies are necessary for a better understanding of the accumulation and effects of PBTs in wild insects, our study clearly demonstrates that long-range transported PBTs have to be taken into account as a relevant driving factor for the decline of insect populations including in remote areas. Our results show that “the contamination of man’s total environment with substances of incredible potential for harm” (Rachel Carson, Silent Spring 1961) is prevailing also in wild insects of near natural habitats. This indicates for legacy contaminants such as PCBs as well es newly introduced chemicals such as PFASs that our actual chemicals legislation is not suited to safeguard a safe operating space for humanity. There is an urgent need to replace PBT substances with safer and more environmentally friendly alternatives, which includes improvements to the chemical authorisation system as well as greater efforts in integrated pest management and integrated vector management. To fully enforce regulations, better destruction facilities, waste management, and improved monitoring are needed.

## Methods

### Study sites and sampling

**Fig. 4:**
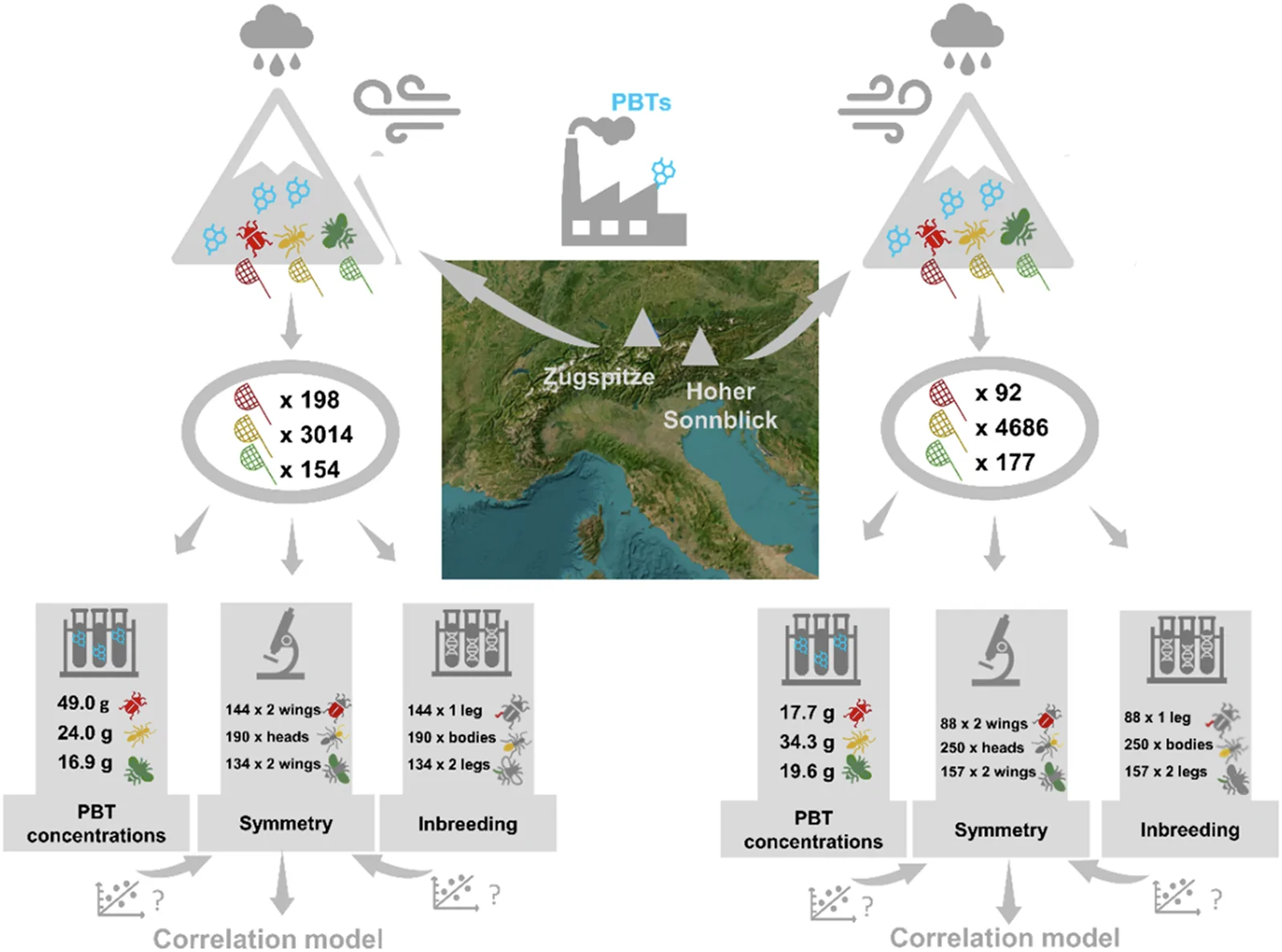
Study design to examine the effects of persistent, bioaccumulative, and toxic chemicals (PBTs) in Alpine insects of different trophic levels. Bumblebees, ants, and carrion beetles as insects of three different trophic levels (herbivores, omnivores, necrophages) were collected at Hoher Sonnblick (Austria) and Zugspitze (Germany), where PBTs enter via atmospheric transport. The number of collected individuals per study site, the biomass of the species analysed with chemical analysis, and the number of morphological and genetic analysed individuals are given. Data on PBT concentrations in the insect bodies were correlated with data on the symmetry of body structures and inbreeding values. llustrations were created in PowerPoint (Microsoft 365) with icons.

Insects were sampled at an altitude of 1600–1900 m above sea level in mountain pine vegetation (*Pinus mugo*). The study sites are located in the vicinity of the environmental research stations Sonnblick Observatory at Hoher Sonnblick (annual precipitation: 1829 mm; average temperature: −5.7 °C) in the Austrian Alps and Schneefernerhaus at Zugspitze (annual precipitation: 1987 mm; average temperature: −3.7 C°) in the German Alps (Fig. 4). At both study sites, atmospheric concentrations of PBTs have been continuously monitored since 2005. Detailed information about monitoring and sampling sites are given in Umweltbundesamt GmbH^38^ and Hierlmeier et al.^25^.

Five terrestrial insect species of three trophic levels were included: Workers of two bumblebee species (*B. pratorum* and *B. wurflenii*) representing the herbivore trophic level, feeding on pollen and nectar; workers of two ant species (*F. aquilonia* and *F. exsecta*), representing an intermediate trophic level being omnivores^78^; and females and males of carrion beetles (*Nicrophorus spp*.) representing a high trophic level by feeding on the carcasses of various vertebrates (necrophages).

Insects were collected between July and September in 2019. For sampling methods of bumblebees and ants, and the sample processing see Hierlmeier et al.^25^. Carrion beetles were sampled using pitfall traps with decaying meat as bait that was not accessible, to avoid contamination of the samples. Due to low sample yields for carrion beetles at Hoher Sonnblick in 2019, additional samples were taken in July 2020. We collected 126 worker bees of *B. pratorum* at Hoher Sonnblick and 83 individuals at Zugspitze. Fifty-one worker bees of *B. wurflenii* were collected at Hoher Sonnblick and 71 at Zugspitze. Five ant nests of *F. aquilonia* were sampled at both study sites, using 30 individuals per nest for detailed analysis. Of *F. exsecta*, five nests were sampled at Hoher Sonnblick, at Zugspitze just two nests due to the limited number of ant nests. Twenty individuals per nest were used for detailed analyses of body morphology and genetics. 6018 (estimated) individuals of *F. aquilonia* and 1701 (estimated) *F. exsecta* were collected for chemical analysis resulting in a biomass of 24.7 and 23.4 g for *F. aquilonia* and 9.6 and 0.6 g of *F. exsecta* at Hoher Sonnblick and Zugspitze, respectively. Ninety-two individuals of *N. vespilloides* from Hoher Sonnblick and 147 from Zugspitze were sampled for chemical, morphological, and genetic analysis. Fitfty-one individuals of *Nicrophorus* spp. were added to the chemical analysis of the samples from the Zugspitze to obtain a higher biomass for further tests (Fig. 4, Supplementary Table 3).

### Sample preparation and chemical analysis

Prior to chemical analysis, both pairs of forewings and two legs from each individual of bumblebees and carrion beetles were pinched off and stored separately in 96% ethanol p.a. for morphological and genetic analysis, respectively. The heads of the ants, intended for morphological analysis, were removed from the mesosoma via scalpel, fixated on standardised pieces of mounting paper, and dried for 24 h. Bodies were stored in 96% ethanol p.a. for genetic analysis.

For chemical analysis, bumblebees and carrion beetles were pooled with respect to species and sampling location. The number of individuals per pooled sample, the wet weight, and the pollutants analysed per pool are given in Supplementary Table 3. The whole bodies of the ants that were additionally sampled from the same nests, were pooled by species (Supplementary Table 3). To avoid any loss of the small and very light parts of the insects, the pooled samples were overlaid with water and homogenised with an ultraturax dispersing instrument. The obtained slurry was freeze-dried, and analysed subsequently by the accredited laboratory of the Environment Agency Austria in Vienna for mercury, brominated flame retardants (BFRs), polychlorinated biphenyls (PCBs), per- and polyfluoroalkyl substances (PFASs), and organochlorine pesticides (OCPs). A total of 46 substances for *B. pratorum* and *B. wurflenii* (Supplementary Data 1A), a total of 46 substances for *F. aquilonia* and *F. exsecta* (Supplementary Data 1B) and a total of 79 substances for *Nicrophorus* spp. (Supplementary Data 1C), were analysed.

The concentrations of 28 BFR congeners (Supplementary Data 1) and six PCB congeners (non-dioxinlike or NDL-PCBs) in insects were analysed. The analytic method for BFRs is in accordance with method EPA 1614, the method for PCBs in accordance with EPA 1668, both published by the U.S. Environmental Protection Agency^79,80^. For the sample preparation, an amount of freeze dried sample equivalent to 3 g wet weight was spiked with ^13^C-labelled surrogate standards followed by Soxhlet extraction with toluene. The extract was evaporated to near-dryness (100 µl of dodecane as keeper), in 9 ml of n-hexane and 1 ml of ethylacetate and added onto the AgNO_3_-Silica gel column of an automatic 4 step (AgNO_3_-silica-gel, H_2_SO_4_-silica-gel, carbon, aluminium) liquid column cleanup system (GO-2HT, Myura, Japan). The BFRs and NDL-PCBs were collected in the fraction eluted from the aluminium column, except DBDPE, which was collected in the fraction from the carbon column. After the transfer to a conical vial, the solution was evaporated under a nitrogen stream to a volume of 100 µl. A ^13^C-labelled injection standard was added, and the solution was transferred into an autosampler vial with a conical insert. The measurements were carried out with a gas-chromatograph coupled to a XEVO APGC-MS/MS (Waters) for BFRs and a gas-chromatograph coupled to a MAT 95 XP high resolution mass spectrometer (Thermo Scientific). For the separation of BFRs a ZB-SemiVolatiles (Phenomenex) 30 m, 0.25 mm, 0.25 µm was used, for the separation of PCBs a DBDIOXIN (J&W) 60 m, 0.25 mm, 0.18 µm. The injector for both columns was a PTV in solvent vent mode. The quantification was done in accordance with an isotope dilution method. The respective limits of detection (LOD) and limits of quantification (LOQ) of the analysed BFR and PCB congeners of the individual pooled samples are listed in Supplementary Data 1.

For the analysis of 12 different PFASs, an amount of 0.62 to 1 g of freeze-dried sample was spiked with an isotope-labelled surrogate standard mixture and extracted with a mixture of 9 ml of acetonitrile and 1 ml 0.5 M formic acid in an ultrasonic bath. The extract was evaporated to 0.5 ml, and 25 mg of EnviCarb powder was added for purification. After 1 min of intense shaking, the extract was centrifuged for 30 min. The supernatant was transferred into an autosampler vial. The extracts were measured on a Waters LC-MS/MS system comprising an Acquity I-Class UPLC and a Xevo TQ-S mass spectrometer. Chromatographic separation was performed on 100 × 2.1 mm Acquity UPLC BEH C18 column (Waters), and an additional Waters Isolator column was used to reduce the instrumental blank values. A gradient elution was performed using Milli-Q water/methanol 98/2 v/v modified with 2 mM ammonium acetate and methanol as eluents. External calibration levels ranged between 0.01 and 10 ng/ml. A sample volume of 2 µl was injected into the system. Procedural blanks were subtracted from each batch, and the concentrations found were corrected with the recoveries of the corresponding isotopically labelled standard. LODs and LOQs of the individual PFAFs per pooled sample are presented Supplementary Data 1.

For the analysis of 32 different OCPs, an amount of 2 g of freeze-dried sample was spiked with an isotope-labelled surrogate standard mixture and extracted by Soxhlet extraction with n-Hexane. The extract was measured with a GC-MS/MS (Agilent) after a two step (Florisil, silica) liquid column cleanup. For the gaschromatographic separation, a DB5-MS (J&W) 60 m, 0.25 mm, 0.25 µm was used. The quantification was done in accordance with an isotope dilution method. LODs and LOQs of individual OCPs per pooled sample are given in Supplementary Data 1.

Aproximately 0.2 g sample was analysed by atomic fluorescence spectroscopy (EN ISO 17852) after microwave assisted aqua regia digestion (ÖNORM EN 13805). LODs and LOQs per pooled sample are given in Supplementary Data 1.

### Correlations between the trophic level and concentrations of PBTs

Correlations between the trophic level and the concentrations of PBTs found in the insect bodies were verified with literature data of nitrogen stable-isotopes δ15N. For bumblebees, we used the data of Chikaraishi et al.^81^, indicating a δ15N value of 2.15‰. For *F. aquilonia* and *F. exsecta*, an average δ15N value of 4.15‰ for both species^82,83^, and for *N. vespilloides* a δ15N value of 6.0‰ indicated by Oelbermann und Scheu^84^ was assumed. The nitrogen stable-isotopes data were correlated with the concentrations of pentabromotoluene, mercury, PCB153, PFNA as representatives of BFRs, heavy metal, PCBs, and PFASs of a pooled sample per organism and study site. Simple linear regression was performed with R (v.4.5.2) and visualised with the package “ggplot2” using the mean of the measured value of pentabromotoluene, mercury, PCB153 and PFNA. To verify the correlations, found between the concentrations of PBTs in the insect bodies and the trophic levels, we performed a Spearman’s rank correlation, using R (v.4.5.2). Here, trophic levels were represented by ranks.

### Landmark-based geometric analysis

Based on previous studies, appropriate body structures and landmarks for the geometric analysis of individual organisms were selected^22,85–87^. Practical constraints had to be considered in the choice of body parts – heads and other body parts would have been difficult to analyse in bumblebess because of their hairiness. Otherwise, wings would have been difficult to analyse in ants given that sampling of winged sexuals is much less practical than sampling wingless, non-reproductive workers from their nests. Technical details on the photography of body structures and setting of landmarks are given in Hierlmeier et al.^25^. To exclude methodological distortion through photographical settings (imaging error), methodological errors due to placing landmarks (digitising error), and to reaffirm the reliability of the data generated in the landmarking process, 20 individuals of *B. pratorum* of each sampling site, 20 individuals of each ant species per region and 10 individuals per region of *N. vespilloides* were photographed and digitised twice to verify imaging and digitising errors as suggested by Klingenberg^22^. The Procrustes ANOVA (*α* = 0.05) for the landmarking validation revealed no statistically significant error concerning digitising or imaging for all organisms. The variation, indicated as F-values and the mean sum of squares, explained by the image setting and the landmarking manner, was consistently lower than that established by differences among individuals or FA-values (Supplementary Table 4).

The detached forewings of bumblebees were photographed from above and from below for quality assurance of the positioning. The images of the wings photographed from below were mirrored, so that all photos faced the same direction to prevent biases in landmarking. Because of the object symmetry (bilateral) of the ant heads, landmarks were set twice for ants^22^. Forewings of *N. vespilloides*, were placed on a microscope slide and photographed from above. Half of the image set was mirrored so that the direction of the wings coincided. Manual landmarking was conducted by tpsdig2 v2.31 positioning the landmarks at distinctive points of the forewing pattern typical for *N. vespilloides*. Landmarks were set twice to calculate residual measurement error in the Procrustes ANOVA.

In total, 20 landmarks were set for bumblebees (Supplementary Fig. 4A), 29 landmarks for ants (Supplementary Fig. 4B, C), and 23 for carrion beetles (Supplementary Fig. 4D). The dataset generated was analysed with the software MorphoJ v1.07a. With a Procrustes fit, variation in shape due to different scales and orientation of the raw landmark data was removed, and aligned coordinates for each individual photo were created. The left and right side of the body structures were compared with a Procrustes ANOVA (*α* = 0.05). Thirty individuals of *B. pratorum* and ten individuals of *B. wurflenii*, as well as seven individuals of carrion beetles with missing landmarks were excluded from further evaluation. In total, 179 *B. pratorum*, 112 *B. wurflenii*, 300 *F. aquilonia*, 140 *F. exsecta*, and 232 *N. vespilloides* individuals were analysed. For carrion beetles, landmarks 1,4, and 21 were excluded from the analysis, as they were missing for many individuals. Fluctuating asymmetry data were extracted, and the index FA2 was calculated at the individual level for the wing shape of bumblebees and beetles and the head shape of ants following Palmer and Strobeck^88^: (R  −  L)/([R  +  L]/2), where R and L are defined as the shape of the right and left side of the individual.

### Microsatellite genotyping and population genetics

DNA was extracted using the QIAGEN Dneasy® Blood and Tissue Kit (Qiagen, Germany) following the manufacturer’s guide, but 50 µl of elution buffer instead of the proposed 200 µl were used for the extraction of DNA of ants and carrion beetles.

Each bumblebee species was genotyped at nine loci, but the loci differed between the species (Supplementary Data 3A). Both species were genotyped at loci BT08, BL11, BT11, BTERN01, and BTERN02^89^. *B. pratorum* was additionally genotyped at loci B96, B100, B124, and B126^90^, and *B. wurflenii* at loci BT10, BT28, BT30, and BL13^89^. Due to problems with the amplification, a high null allele frequency combined with significant deviations from the Hardy-Weinberg equilibrium the loci BTERN01, B100, and B126 were excluded from further analysis for *B. pratorum*. For *B. wurflenii*, BL13 was excluded from further analysis due to a low number of alleles. For the amplification of all loci, a polymerase chain reaction (PCR) formulation was prepared with 0.5 μl of the bumblebee sample DNA, 1 μl 5X OneTaq® Quick-Load® Reaction Buffer, 0.1 μl dNTPs (10 mM), 0.1 μl forward primer (10 μM), 0.1 μl labelled forward primer (10 μM), 0.1 μl reverse primer (10 μM), and 0.025 μl OneTaq® Quick-Load^®^ DNA Polymerase, and 4.5 μl of a reaction dissolution consisting of 3.75 μl Milli-Q® water.

For *F. aquilonia*, 12 loci were used for the genetic analysis, namely FL21^91^, FE7, FE11, FE37, FE42, FE16, FE13, FE49, FE17, FE38^92^, P22^93^, and Fy3^94^, but locus FE49 was excluded due to scoring issues (Supplementary Data 3A). Two PCR setups were prepared: For FL21, FE7, FE37, Fy3, FE42, FE16, FE13, FE49, FE17, and FE38, each reaction mixture of 10 µl reaction volume contained 1 µl of DNA-extract, 5 µl of 2x Rotor-Gene Master Mix (Qiagen), 0.02 µl of 10 µM M13-tailed forward primer, 0.2 µl of 10 µM fluorescent labelled M13 primer, 0.2 µl of 10 µM untailed reverse primer, and 3.58 µl Milli-Q® water. For FE11 and P22, each reaction mixture of identical volume contained the same setup of DNA extract and primers as used for the other microsatellites. However, instead of 2x Rotor-Gene Master Mix (Qiagen), 2 µl of 5x One Taq® Quick-Load® Reaction Buffer (New England Biolabs) with 0.2 µl of 10 mM dNTPs and 0.05 µl of One Taq Quick-Load® DNA polymerase was used with 6.33 µl of Milli-Q® for volume completion.

For *F. exsecta*, 12 loci were used for the genetic analysis, namely, FL21^91^, FE7, FE11, FE13, FE15, FE42, FE38, FE37, FE49, FE51^92^, P22^93^, and Fy3^94^ (Supplementary Data 3A). As FE11 and Fy3 were monomorphic in both regions, they were excluded. The same PCR setups as described for *F. aquilonia* were used. For Fy3, FE38, FE37, and FE51, the 2x Rotor-Gene Master Mix (Qiagen) was used. FL21, FE7, FE11, FE13, FE15, FE42, FE49, and P22, were analysed using 5x One Taq® Quick-Load® Reaction Buffer (New England Biolabs), dNTPs and One Taq® Quick-Load® DNA polymerase.

For *N. vespilloides*, 12 loci were used for genetic analyses, namely Nvesp_A, Nvesp_D, Nvesp_J, Nvesp_M, Nvesp_H, Nvesp_B, Nvesp_F, Nvesp_I, Nvesp_P, Nvesp_E, Nvesp_G, and Nvesp_O^95^ (Supplementary Data 3A). The setup for the OneTaq® Quick-Load® DNA polymerase (New England Biolabs) was used for all microsatellites as described for the ant species above.

The PCRs were carried out on a UnoCycler (VWR), PCR settings is given in Supplementary Data 3B. Fragment analysis was carried out on an ABI3730XL genetic analyser (Applied Biosystems) by a commercial provider (Comprehensive Cancer Centre DNA Sequencing & Genotyping Facility, University of Chicago, USA). The sequenced DNA fragments were visualised with GeneMarker® v.3.0.1 (SoftGenetics, State College, PA, USA). To verify the suitability of the markers, linkage disequilibrium (LD), and deviations from the Hardy-Weinberg equilibrium were tested. With a Bonferroni-Holm correction for multiple testing, false-positive outputs of LD and the Hardy-Weinberg-Equilibrium were minimised. F-statistics were calculated and microsatellite null alleles were tested. Based on the observed heterozygosity, the numbers of expected heterozygotes under the Hardy-Weinberg-Equilibrium were generated. The multilocus heterozygosity (MLH) was calculated as an indicator for the genetic status of inbreeding at the individual level. Analysis of the genetic data of bumblebees was done in R (v.4.0.3), using the package “genepop”. Calculations were performed with the “inbreedR” package. The analyses of ants and carrion beetles were done in GenAlEx v.6.503 and Arlequin v.3.5.2.2. One-tailed t-tests were conducted between H_e_ and H_o_ in Excel version 2207 using Analysis ToolPak.

### Statistical evaluation of correlations

Simple linear regression was performed with R (v.4.4.1) to investigate whether FA values increase with the PBT concentrations over all species. Individuals with missing FA-scores were excluded from these evaluations as well as individuals with >0.2, which were considered as outliers (Supplementary Table 2).

Potential correlations between PBT concentrations, fluctuating asymmetry, and inbreeding within species, were tested with typal multiple linear models (MLM) in R (v.4.4.1) using the packages “lmtest“, “vegan“, “ggplot2“and “performanceEstimation“. The FA2 index (FA, fluctuating asymmetry) was set as the dependent variable, and the concentration of PBTs and the inbreeding coefficient MLH as independent variables. If a substance was detected in an organism for one study site but was missing in the other study site, the value which corresponded to the limit of quantification (LOQ) in the gas chromatography/high-resolution mass spectrometry analysis was used as value in the study site with missing data. It can be assumed that the respective PBTs are present in the species but just slightly below the (LOD). Model fit was checked with the R package “DHARMa”. Where residual analysis showed poor model fits, a logarithmic transformation was done to achieve a reasonable normal distribution of the residuals. Shapiro-Wilk and Breusch-Pagan tests were performed to test for normal distribution and heteroskedasticity, respectively.

To take into consideration chemical measurement inaccuracy of +/-30% for organic PBTs^96^ and +/-13% for mercury^97^, Monte Carlo Simulations were conducted and included in the original linear models with at-random altered substance concentrations within the range of measurement inaccuracy testing for model output robustness. Simulations were done with 1000 Monte Carlo runs, based on a pilot study (data not shown), in which a series from 20 to up to 10,000 Monte Carlo runs were tested. The pilot studies showed that after 200 to 500 runs, medians of the *p*-values and the median R^2^-values stabilised with less than 10% differences from the results for 1000 and 2000 runs. If a model’s median *p*-value (*α* = 0.05) remained significant after 1000 Monte Carlo Simulation runs, its original result was accepted and interpreted. Otherwise, the original model was rejected.

### Statistics and reproducibility

No statistical method was used to predetermine sample size. All statistical analyses of the data were were performed in in the R statistical environment (v.4.0.3; v.4.4.1; v.4.5.2). Analysis of the genetic data of ants and carrion beetles was also done using GenAlEx v.6.503 and Arlequin v.3.5.2.2. MorphoJ v1.07a was used for landmark-based geometric analysis. All statistical analyses are described in detail in the relevant methods subsections. Data points are shown on the figures, and the number of samples is given in the corresponding figure legends. Each individual represents an independent biological replicate. In a pilot study, FA of 40 bumblebees, 80 ants and 20 carrion beetles were used as technical replicates for quality assurance.

### Reporting summary

Further information on research design is available in the Nature Portfolio Reporting Summary linked to this article.

## Supplementary information

Supplementary Information

Description of Additional Supplementary Files

Supplementary Data 1

Supplementary Data 2

Supplementary Data 3

Reporting Summary

## Data Availability

The source data for the graphs can be found in Supplementary Data 1 and 2. All other data are available from the corresponding author on reasonable request.
