# Supplementary material for: Trophic-level dependent concentrations of forever chemicals in insects of pristine areas are linked to morphological changes
Source: Commun Biol. 2026 Jul 9;9:1441. doi: 10.1038/s42003-026-10586-1 (PMC13724735; doi:10.1038/s42003-026-10586-1)
Supplement: Supplementary file 1 [file 42003_2026_10586_MOESM1_ESM.pdf]

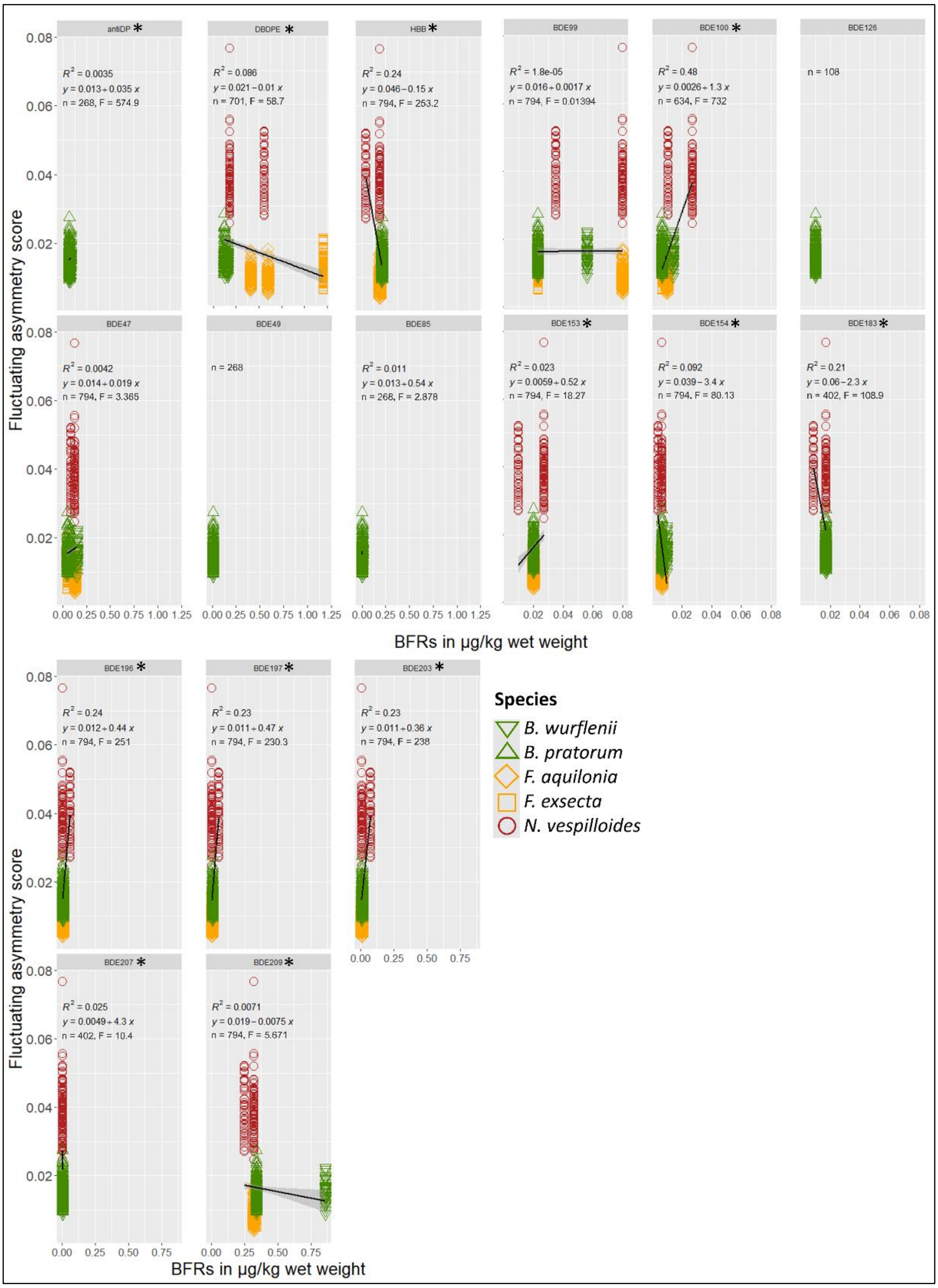

Supplementary Figure 1: **Correlation of brominated flame retardants (BFRs) with fluctuating asymmetry (FA) scores of bumblebees, ants, and carrion beetles.** Correlations between the concentrations of 23 brominated flame retardants (BFRs) and fluctuating asymmetry scores of the individuals found in *Bombus wurflenii, Bombus pratorum, Formica aquilonia, Formica exsecta*, and *Nicrophorus vespilloides*. *indicate significantly positive or negative correlations between BFR concentration and FA scores, n = number of data points, F = F-value indicating variation.

.

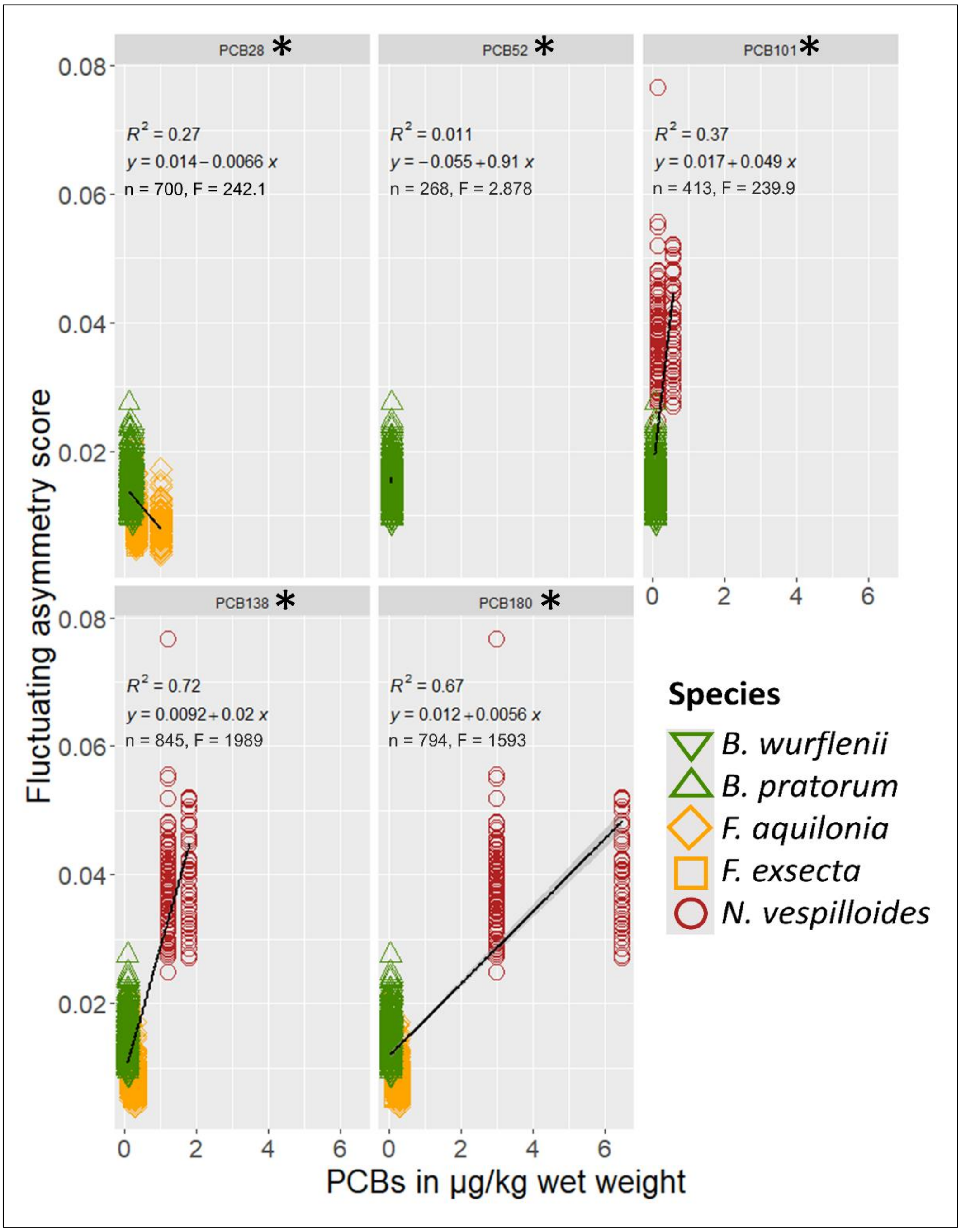

Supplementary Figure 2: **Correlation of polychlorianted biphenyls (PCBs) with fluctuating asymmetry (FA) scores of bumblebees, ants, and carrion beetles.** Correlations between the concentrations of five polychlorianted biphenyls (PCBs) and fluctuating asymmetry scores of individuals found in *Bombus wurflenii, Bombus pratorum, Formica aquilonia, Formica exsecta*, and *Nicrophorus vespilloides*. *indicate significantly positive or negative correlations between PCB concentration and FA scores, n = number of data points, F = F-value indicating variation.

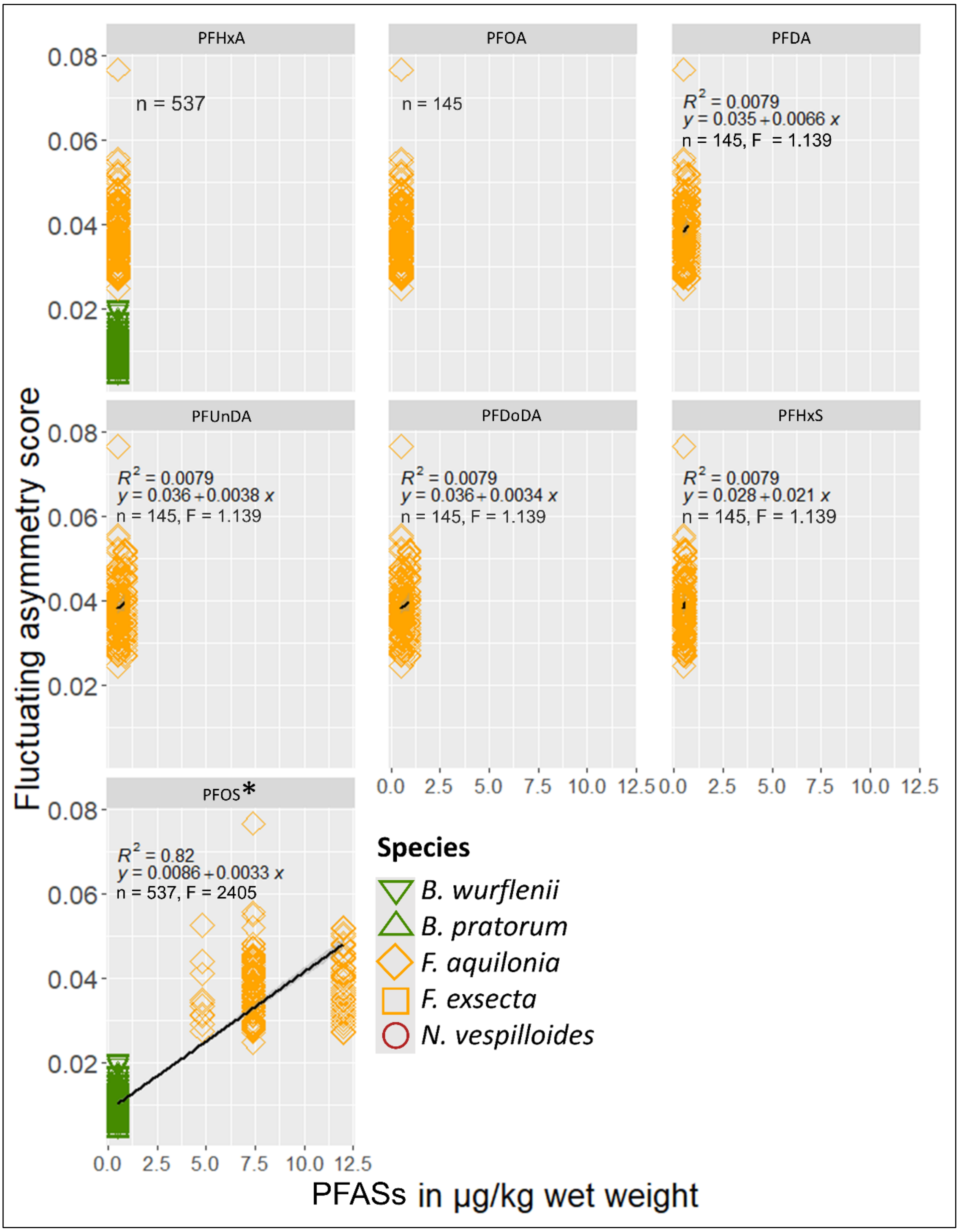

Supplementary Figure 3: **Correlation of per- and polyfluoroalkyl substances (PFASs) with fluctuating asymmetry (FA) scores of bumblebees, ants, and carrion beetles.** Correlation between the concentrations of seven per- and polyfluoroalkyl substances (PFASs) and fluctuating asymmetry scores of the individuals found in *Formica aquilonia, Formica exsecta,* and *Nicrophorus vespilloides*. *indicate significantly positive or negative correlations between PFAS concentration and FA scores, n = number of data points, F = F-value indicating variation.

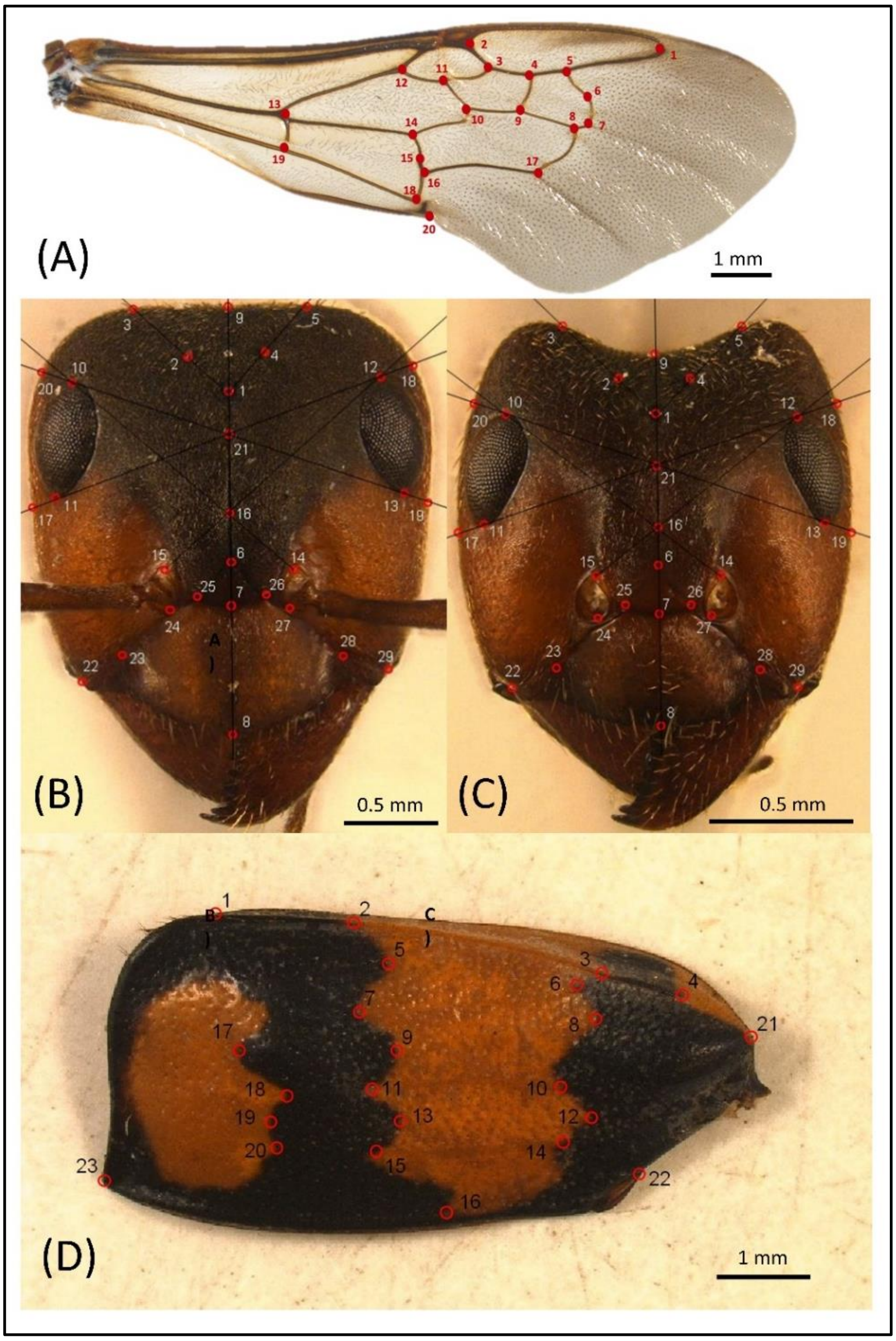

Supplementary Figure 4: **Morphological parameters and landmarks used to evaluate fluctuating asymmetry in bumblebees, ants, and carrion beetles.** Red dots indicate the positions of the landmarks used. (A) Twenty landmarks were positioned at the nadirs of the distal contour lines of vein intersections of *Bombus wurflenii* and *Bombus pratorum*. On distinctive points on the heads of *Formica aquilonia* (B) and *Formica exsecta* (C), 29 landmarks were positioned with the help of auxiliary lines. At distinctive points of the forewing pattern typical for (D) *Nicrophorus vespilloides*, 23 landmarks were positioned. (A), (B), (C) were created by the authors, (D) was provided with permission by Joelle Kröll.

Supplementary Table 1: **Scores of fluctuating asymmetry and Procrustes analyses of variance testing for differences between the left and right shape of body structures found in bumblebees, ants, and carrion beetles.** Ranges of fluctuating asymmetry scores (FA-scores) and Procrustes analyses of variance testing for differences among individuals and wing shape fluctuating asymmetry in *Bombus wurflenii*, *Bombus pratorum*, and *Nicrophorus vespilloides* as well as head shape of *Formica aquilonia* and *Formica exsecta*. SS, sum of squares; MS, mean sum of squares; df, degrees of freedom; F, F-value indicating variation; p, p-value.

Species	Object	Effect	SS	MS	df	F	p	FA-scores
Bombus pratorum	forewings	Individual	314.1 $\times 10^{-3}$	51.93 $\times 10^{-6}$	6048	3.05	<0.0001	0.009 – 0.029
		Fluctuating asymmetry	192.95 $\times 10^{-3}$	17.02 $\times 10^{-6}$	6048	1.32	<0.0001	
Bombus wurflenii	forewings	Individual	161,42 $\times 10^{-3}$	40.76 $\times 10^{-6}$	3960	6.89	<0.0001	0.009 – 0.022
		Fluctuating asymmetry	23.44 × 10-3	5.92 $\times 10^{-6}$	3960	6.64	<0.0001	
Formica aquilonia	head	Individual	295.6 x 10^{-3}	36.99 $\times 10^{-6}$	7992	5.52	<0.0001	0.004 – 0.032
		Fluctuating asymmetry	53.50 $\times 10^{-3}$	6.70 $\times 10^{-6}$	7992	2.35	<0.0001	
Formica exsecta	head	Individual	185.44 $\times 10^{-3}$	49.41 $\times 10^{-6}$	3753	4.34	<0.0001	0.005 – 0.029
		Fluctuating asymmetry	42.76 $\times 10^{-3}$	11.39 $\times 10^{-6}$	3753	2.10	<0.0001	
Nicrophorus vespilloides	forewings	Individual	1.99	253.03 $\times 10^{-6}$	7848	12.22	<0.0001	0.025 – 0.077
		Fluctuating asymmetry	162.52 $\times 10^{-3}$	20.71 $\times 10^{-6}$	7848	0.14	<0.0001	

Supplementary Table 2: **Estimates of genetic diversity**. The Analysis is based on microsatellite loci in *Bombus pratorum, Bombus wurflenii, Formica aquilonia, Formica exsecta and Nicrophorus vespilloides* including allelic richness (NA), expected heterozygosity (H_e), observed heterozygosity (H_o), and inbreeding coefficient (F_{IS}).

		**Hoher Sonnblick**				**Zugspitze**			
**Species**	**Locus**	**N_A**	**H_e**	**H_o**	**F_{IS}**	**N_A**	**H_e**	**H_o**	**F_{IS}**
Bombus pratorum	B96⁺	16	0.90	0.83	0.08	16	0.89	0.82	0.08
	BL11⁺	25	0.92	0.90	0.07	19	0.69	0.93	-0.02
	B124	10	0.76	0.70	0.03	10	0.76	0.66	0.13
	BT08*	13	0.82	0.72	0.12	10	0.85	0.85	0.00
	BT11	17	0.84	0.79	0.06	16	0.81	0.84	-0.04
	BTERN02⁺	25	0.80	0.68	0.15	24	0.89	0.88	0.01
**Overall**		**17.67**	**0.84**	**0.77**	**0.77**	**15.83**	**0.82**	**0.83**	**0.03**
Bombus wurflenii	BL11⁺	20	0.92	0.90	0.02	19	0.91	0.93	-0.01
	BT30	7	0.67	0.70	-0.05	7	0.56	0.63	-0.12
	B10	17	0.94	1.00	-0.07	21	0.94	0.89	0.06
	BT28	2	0.31	0.38	-0.21	2	0.28	0.33	-0.18
	BT11	7	0.83	0.84	-0.02	8	0.83	0.70	0.16
	BTERN01⁺	19	0.92	0.90	0.02	23	0.94	0.95	-0.01
	BTERN02⁺	17	0.88	1.00	-0.07	19	0.94	0.81	0.14
	BT08	22	0.95	0.81	0.15	19	0.91	0.79	0.14
**Overall**		**13.88**	**0.80**	**0.82**	**-0.03**	**15.83**	**0.79**	**0.75**	**0.02**
Formica aquilonia	FL21	6	0.76	0.78	-0.02	9	0.79	0.75	-0.05
	FE7	6	0.57	0.49	0.15	7	0.77	0.71	0.07
	FE11	4	0.71	0.78	-0.10	4	0.53	0.52	-0.02
	FE37	6	0.70	0.37	0.48	6	0.74	0.81	-0.099
	Fy3	3	0.29	0.32	-0.09	3	0.58	0.66	-0.14
	FE42	4	0.52	0.32	0.37	2	0.50	0.56	-0.12
	FE16	8	0.67	0.43	0.36	7	0.75	0.26	0.66
	FE13	4	0.60	0.62	-0.03	4	0.69	0.66	0.04
	P22	3	0.07	0.06	0.16	4	0.47	0.46	0.022
	FE17	5	0.59	0.41	0.30	2	0.21	0.21	0.00
	FE38	3	0.56	0.50	0.10	2	0.47	0.49	-0.04
**Overall**		**4.10**	**0.54**	**0.62**	**0.15**	**4.55**	**0.050**	**0.55**	**0.04**
Formica exsecta	FL21	7	0.76	0.99	-0.30	2	0.45	0.70	-0.54
	FE7	7	0.82	0.97	-0.18	3	0.58	0.43	0.27
	FE13	4	0.55	0.60	-0.09	3	0.66	0.74	-0.19
	FE15	3	0.27	0.20	0.27	3	0.60	0.73	-0.23
	FE42	4	0.60	0.70	-0.17	2	0.12	0.13	-0.07
	FE38	3	0.62	0.67	-0.08	2	0.40	0.50	-0.25
	FE37	3	0.27	0.31	-0.13	2	0.46	0.28	0.41
	FE49	2	0.34	0.43	-0.27	3	0.56	0.80	-0.44
	P22	4	0.61	0.51	0.17	2	0.47	0.75	-0.60
	FE51	4	0.54	0.77	-0.44	3	0.50	0.73	-0.44
**Overall**		**4.10**	**0.54**	**0.62**	**-0.12**	**2.50**	**0.050**	**0.58**	**-0.20**
Nicrophorus vespilloides	Nvesp_D	8	0.82	0.79	0.04	8	0.83	0.72	0.13
	Nvesp_M	10	0.85	0.85	0.00	11	0.84	0.83	0.02
	Nvesp_H*	15	0.86	0.85	0.02	15	0.86	0.78	0.10
	Nvesp_F	9	0.80	0.66	0.18	10	0.83	0.83	0.00
	Nvesp_I*	6	0.63	0.58	0.08	8	0.59	0.51	0.14
	Nvesp_P	10	0.80	0.73	0.09	11	0.82	0.69	0.16
	Nvesp_E	9	0.82	0.67	0.18	10	0.84	0.74	0.13
**Overall**		**9.57**	**0.80**	**0.73**	**0.08**	**10.43**	**0.80**	**0.73**	**0.10**

Supplementary Table 3: **Overview of individuals of bumblebees, ants, and carrion beetles, analysed for the effects of persistent, bioaccumulative, and toxic chemicals.** Location, species, number of individuals, and biomass per pooled sample for chemical analyses of PBTs and number of morphological and genetically analysed individuals as well as the number of species included in statistical evaluation. For the estimation of individuals of ants, the weight of 0.008 g and 0.006 g for *F. aquilonia* and for *F. exsecta* per individual was used, respectively. BFRs = brominated flame retardants, mercury, OCPs = organochlorine pesticides, PCBs = polychlorinated biphenyls, PFASs = per- and polyfluoroalkyl substances.

**Sample code**	**Location**	**Species**	**No. of individuals**	**Weight (g)**	**PBT measured**	**No. of individuals morphological and genetically analysed**	**No. of individuals included in statistical evaluation of correlations**
Pool 1	Hoher Sonnblick	*Bombus pratorum*	126	14.0	BFRs, mercury, PCBs, PFASs	111	168
Pool 2	Zugspitze	*Bombus pratorum*	83	9.1	BFRs, mercury, PCBs, PFASs	68	
Pool 3	Hoher Sonnblick	*Bombus wurflenii*	51	5.6	BFRs, mercury, PCBs, PFASs	46	111
Pool 4	Zugspitze	*Bombus wurflenii*	71	7.8	BFRs, mercury, PCBs, PFASs	66	
Pool 5	Hoher Sonnblick	*Formica aquilonia*	1525[1] (estimated)	12.2	BFRs, PCBs		
Pool 6	Zugspitze	*Formica aquilonia*	13631 (estimated)	10.9 g	BFRs, PCBs		
Pool 7	Hoher Sonnblick	*Formica aquilonia*	313[2] (estimated)	2.5	mercury, PFASs	30	297
Pool 8	Hoher Sonnblick	*Formica aquilonia*	313[2] (estimated)	2.5	mercury, PFASs	30	
Pool 9	Hoher Sonnblick	*Formica aquilonia*	313[2] (estimated)	2.5	mercury, PFASs	30	
Pool 10	Hoher Sonnblick	*Formica aquilonia*	313[2] (estimated)	2.5	mercury, PFASs	30	
Pool 11	Hoher Sonnblick	*Formica aquilonia*	313[2] (estimated)	2.5	mercury, PFASs	30	

Pool 12	Zugspitze	*Formica aquilonia*	313[2] (estimated)	2.5	mercury, PFASs	30	
Pool 13	Zugspitze	*Formica aquilonia*	313_2 (estimated)	2.5	mercury, PFASs	30	
Pool 14	Zugspitze	*Formica aquilonia*	313[2] (estimated)	2.5	mercury, PFASs	30	
Pool 15	Zugspitze	*Formica aquilonia*	313[2] (estimated)	2.5	mercury, PFASs	30	
Pool 16	Zugspitze	*Formica aquilonia*	313[2] (estimated)	2.5	mercury, PFASs	30	
Pool 17	Hoher Sonnblick	*Formica exsecta*	1243[3] (estimated)	7.5	BFRs, PCBs, PFASs		
Pool 18	Hoher Sonnblick	*Formica exsecta*	150[4] (estimated)	0.9	mercury	20	
Pool 19	Hoher Sonnblick	*Formica exsecta*	83[4] (estimated)	0.5	mercury	20	
Pool 20	Hoher Sonnblick	*Formica exsecta*	100[4] (estimated)	0.6	mercury	20	
Pool 21	Hoher Sonnblick	*Formica exsecta*	17[4] (estimated)	0.1	mercury	20	140
Pool 22	Hoher Sonnblick	*Formica exsecta*	8[4] (estimated)	0.05	mercury	20	
Pool 23	Zugspitze	*Formica exsecta*	33 (estimated)	0.2	mercury	20	
Pool 24	Zugspitze	*Formica exsecta*	67 (estimated)	0.4	mercury	20	
Pool 25	Hoher Sonnblick	*Nicrophorus vespilloides*	25	3.9	mercury, PFASs	22	
Pool 26	Hoher Sonnblick	*Nicrophorus vespilloides*	67	14.0	BFR, mercury, PCBs, PFASs	66	148
Pool 27	Zugspitze	*Nicrophorus spp.*	198	49.0	BFRs, mercury, PCBs, PFASs, OCPs	144[5]	

[1]consisting of the individuals from pools 5-8 and 12-16, respectively
[2]After removal of individuals for pool 5 and 11
[3]consisting of the individuals from pools 18-22
[4]After removal of individuals for pool 17
[5]only individuals of *N. vespilloides* were analysed

Supplementary Table 4: **Validation results for landmarking of forewings of bumblebees and carrion beetles as well as ant heads to compare biological and technical variation.** No statistically significant error concerning digitizing or imaging for all organisms during landmarking in fluctuating asymmetry analysis. df= degrees of freedom.

Organism	Effect	Sum of squares	Mean squares	df	F	p
Bombus pratorum **Hoher Sonnblick**	Individual	4.98×10^{-2}	8.15×10^{-5}	612	2.43	<0.0001
	Imaging error	2.42×10^{-2}	1.98×10^{-5}	1224	0.94	<0.8753
	Digitising error	4.48×10^{-2}	2.10×10^{-5}	2268		
Bombus pratorum **Zugspitze**	Individual	4.62×10^{-2}	7.14×10^{-5}	648	5.88	<0.0001
	Imaging error	1.25×10^{-3}	9.36×10^{-7}	1332	1.24	<0.0001
	Digitising error	1.92×10^{-3}	7.52×10^{-2}	2556		
Formica aquilonia	Individual	2.21×10^{-1}	1.05×10^{-4}	2106	8.09	<0.0001
	Imaging error	2.80×10^{-2}	1.30×10^{-5}	2160	9.95	<0.0001
	Digitising error	5.65×10^{-3}	1.30×10^{-6}	4320		
Formica exsecta	Individual	2.21×10^{-1}	1.05×10^{-4}	2106	5.21	<0.0001
	Imaging error	4.36×10^{-2}	2.02×10^{-5}	2160	6.52	<0.0001
	Digitising error	1.34×10^{-2}	3.09×10^{-6}	4320		
Nicrophorus vespilloides	Individual	5.55×10^{-1}	6.95×10^{-4}	798	144.32	<0.0001
	Imaging error	4.0×10^{-3}	4.82×10^{-6}	840	0.35	1.0
	Digitising error	6.86×10^{-2}	1.36×10^{-5}	5040		
