# Supplementary material for: Trophic-level dependent concentrations of forever chemicals in insects of pristine areas are linked to morphological changes
Source: Commun Biol. 2026 Jul 9;9:1441. doi: 10.1038/s42003-026-10586-1 (PMC13724735; doi:10.1038/s42003-026-10586-1)
Supplement: Supplementary file 2 [file 42003_2026_10586_MOESM2_ESM.pdf]

Description of Additional Supplementary Files

File name: Supplementary Data 1

Description: The data of PBT concentrations per species, study site as well as the limits of detection and quantification.

File name: Supplementary Data 2

Description: The FA scores of organisms.

File name: Supplementary Data 3

Description: The loci used for genetic analysis and PCR settings
