# Supplementary material for: Trophic-level dependent concentrations of forever chemicals in insects of pristine areas are linked to morphological changes
Source: Commun Biol. 2026 Jul 9;9:1441. doi: 10.1038/s42003-026-10586-1 (PMC13724735; doi:10.1038/s42003-026-10586-1)
Supplement: Supplementary file 3 [file 42003_2026_10586_MOESM6_ESM.pdf]

nature portfolio

Corresponding author(s): Hierlmeier-Hackl, Veronika

Last updated by author(s): Jun 16, 2026

Reporting Summary

Nature Portfolio wishes to improve the reproducibility of the work that we publish. This form provides structure for consistency and transparency in reporting. For further information on Nature Portfolio policies, see our Editorial Policies and the Editorial Policy Checklist.

Statistics

For all statistical analyses, confirm that the following items are present in the figure legend, table legend, main text, or Methods section.

n/a	Confirmed	
☐	☒	The exact sample size (*n*) for each experimental group/condition, given as a discrete number and unit of measurement
☐	☒	A statement on whether measurements were taken from distinct samples or whether the same sample was measured repeatedly
☐	☒	The statistical test(s) used AND whether they are one- or two-sided<br>*Only common tests should be described solely by name; describe more complex techniques in the Methods section.*
☒	☐	A description of all covariates tested
☐	☒	A description of any assumptions or corrections, such as tests of normality and adjustment for multiple comparisons
☐	☒	A full description of the statistical parameters including central tendency (e.g. means) or other basic estimates (e.g. regression coefficient) AND variation (e.g. standard deviation) or associated estimates of uncertainty (e.g. confidence intervals)
☐	☒	For null hypothesis testing, the test statistic (e.g. *F*, *t*, *r*) with confidence intervals, effect sizes, degrees of freedom and *P* value noted<br>*Give P values as exact values whenever suitable.*
☒	☐	For Bayesian analysis, information on the choice of priors and Markov chain Monte Carlo settings
☒	☐	For hierarchical and complex designs, identification of the appropriate level for tests and full reporting of outcomes
☒	☐	Estimates of effect sizes (e.g. Cohen's *d*, Pearson's *r*), indicating how they were calculated

Our web collection on statistics for biologists contains articles on many of the points above.

Software and code

Policy information about availability of computer code

Data collection	No software was used for data collection.
Data analysis	Data analyses were performed in in the R statistical environment (v.4.0.3; v.4.4.1; v.4.5.2); MorphoJ v1.07a was used for landmark-based geometric analysis; analysis of the genetic data (ants and carrion beetles) was done using GenAlEx v.6.503 and Arlequin v.3.5.2.2.

For manuscripts utilizing custom algorithms or software that are central to the research but not yet described in published literature, software must be made available to editors and reviewers. We strongly encourage code deposition in a community repository (e.g. GitHub). See the Nature Portfolio guidelines for submitting code & software for further information.

Data

Policy information about availability of data

All manuscripts must include a data availability statement. This statement should provide the following information, where applicable:

- Accession codes, unique identifiers, or web links for publicly available datasets
- A description of any restrictions on data availability
- For clinical datasets or third party data, please ensure that the statement adheres to our policy

The source data for the graphs can be found in Supplementary Data 1 and 2. All other data are available from the corresponding author on reasonable request.

Research involving human participants, their data, or biological material

Policy information about studies with human participants or human data. See also policy information about sex, gender (identity/presentation), and sexual orientation and race, ethnicity and racism.

Reporting on sex and gender	n/a
Reporting on race, ethnicity, or other socially relevant groupings	n/a
Population characteristics	n/a
Recruitment	n/a
Ethics oversight	n/a

Note that full information on the approval of the study protocol must also be provided in the manuscript.

Field-specific reporting

Please select the one below that is the best fit for your research. If you are not sure, read the appropriate sections before making your selection.

☐ Life sciences ☐ Behavioural & social sciences ☒ Ecological, evolutionary & environmental sciences

For a reference copy of the document with all sections, see nature.com/documents/nr-reporting-summary-flat.pdf

Ecological, evolutionary & environmental sciences study design

All studies must disclose on these points even when the disclosure is negative.

Study description	The concentrations of persistent, bioaccumulative and toxic chemicals (PBTs) in wild Alpine insects of different trophic levels were investigated. We used fluctuating asymmetry (FA) as proxy for fitness and tested correlations between FA and PBT concentrations. Inbreeding as an alternative cause for FA was examined via population genetics.
Research sample	In this study, two species of bumblebees (Bombus pratorum and Bombus wurflenii), two species of ants (Formica exsecta and Formica aquilonia), and carrion beetles (Nicrophorus spp.) were examined. Wild insects were collected in the Bavarian Alps (Zugspitze) and Austrian Alps (Hoher Sonnblick). The organisms were choosen due to their occurence in both study areas. These species represent a herbivore, an intermediate and a high trophic level.
Sampling strategy	Bumblebees were individually caught using Duran® borosilicate glass flasks with ground glass joints, ants were collected using forceps, but also with a motorized suction device to avoid contact with formic acid. Carrion beetles were sampled using pitfall traps with decaying meat as bait. The sample size was based on the biomass required for chemical analysis.
Data collection	PBT concentrations were given by the accredited laboratory; data of FA and genetic analysis were recorded by VHH, PK, SG, CMS, VTS.
Timing and spatial scale	Insects were collected between July and September in 2019. Due to low sample yields for carrion beetles at Hoher Sonnblick in 2019, additional samples were taken in July 2020. Insects were collected at an altitude of 1600 -1900 m above sea level in mountain pine vegetation.
Data exclusions	Some landmarks (morphological analysis) and some loci (genetic analysis) were excluded as they were not suitable for further analysis.
Reproducibility	Data are provided in Supplementary Data 1 and 2 to reproduce the study.
Randomization	Insects were sampled random at the study sites, no transects or similar were included. Insects were pooled with respect to species and sampling location.
Blinding	Blinding was not relevant in this study as an analysis of species and sampling location was intentional.

Did the study involve field work? ☒ Yes ☐ No

Field work, collection and transport

Field conditions	Insects were collected from July to September, on windless days with no precipitation.
Location	Insects were sampled at an altitude of 1600 -1900 m above sea level in mountain pine vegetation (Pinus mugo).
Access & import/export	In the Nationalpark Hohe Tauern, insects were sampled under permit of the Hohe Tauern National Park Administration.
Disturbance	The collection was done over several months to keep the pressure on the populations low. Pitfall traps for carrion beetles were controlled regularly.

Reporting for specific materials, systems and methods

We require information from authors about some types of materials, experimental systems and methods used in many studies. Here, indicate whether each material, system or method listed is relevant to your study. If you are not sure if a list item applies to your research, read the appropriate section before selecting a response.

Materials & experimental systems

n/a	Involved in the study	
☒	☐	Antibodies
☒	☐	Eukaryotic cell lines
☒	☐	Palaeontology and archaeology
☐	☒	Animals and other organisms
☒	☐	Clinical data
☒	☐	Dual use research of concern
☒	☐	Plants

Methods

n/a	Involved in the study	
☒	☐	ChIP-seq
☒	☐	Flow cytometry
☒	☐	MRI-based neuroimaging

Animals and other research organisms

Policy information about studies involving animals; ARRIVE guidelines recommended for reporting animal research, and Sex and Gender in Research

Laboratory animals	The study did not involve laboratory animals.
Wild animals	Two species of bumblebess (Bombus pratorum and Bombus wurflenii), two species of ants (Formica exsecta and Formica aquilonia), and carrion beetles (Nicrophorus spp.) were investigated. Bumblebees were individually caught using Duran® borosilicate glass flasks with ground glass joints, ants were collected using forceps, but also with a motorized suction device to avoid contact with formic acid. Carrion beetles were sampled using pitfall traps with decaying meat as bait and transferred to Duran® borosilicate glass flasks. The flasks with samples were intermediately stored in a cool bag followed by euthanizing the insects without contamination by inserting the flasks in a dryshipper filled with liquid nitrogen (−196 °C).
Reporting on sex	Just diploid workers of bumblebees and ants were used in this study to perform microsatellite genotyping and population genetics.
Field-collected samples	Sampled insects were wrapped in aluminum foil on arrival in the laboratory, transferred into glass tubes, and stored in a freezer at −20 °C.
Ethics oversight	Common insect species only.

Note that full information on the approval of the study protocol must also be provided in the manuscript.

Plants

Seed stocks	n/a
Novel plant genotypes	n/a
Authentication	n/a
